# Analysis of adherent cell culture lysates with low metabolite concentrations using the Biocrates AbsoluteIDQ p400 HR kit

**DOI:** 10.1038/s41598-022-11118-7

**Published:** 2022-05-13

**Authors:** Raphaela Fritsche-Guenther, Yoann Gloaguen, Alina Eisenberger, Jennifer A. Kirwan

**Affiliations:** 1 Berlin Institute of Health (BIH) @ Charité, BIH Metabolomics Platform, Anna-Louisa-Karsch-Str. 2, 10178 Berlin, Germany; 2 Core Unit Bioinformatics, Berlin Institute of Health (BIH) @ Charité, Chariteplatz 1, 10117 Berlin, Germany

**Keywords:** Cancer, Cell biology

## Abstract

The Absolute*IDQ* p400 HR kit is a commercial product for targeted metabolomics. While the kit has been validated for human plasma and serum, adherent cell lysates have not yet been evaluated. We have optimized the detection of polar and lipid metabolites in cell lysates using the kit to enable robust and repeatable analysis of the detected metabolites. Parameters optimized include total cell mass, loading volume and extraction solvent. We present a cell preparation and analytical method and report on the performance of the kit with regard to detectability of the targeted metabolites and their repeatability. The kit can be successfully used for a relative quantification analysis of cell lysates from adherent cells although validated only for human plasma and serum. Most metabolites are below the limit of the Biocrates’ set quantification limits and we confirmed that this relative quantification can be used for further statistical analysis. Using this approach, up to 45% of the total metabolites in the kit can be detected with a reasonable analytical performance (lowest median RSD 9% and 13% for LC and FIA, respectively) dependent on the method used. We recommend using ethanol as the extraction solvent for cell lysates of osteosarcoma cell lines for the broadest metabolite coverage and 25 mg of cell mass with a loading volume of 20 µL per sample.

## Introduction

In vitro cell culture models are a common method to address multiple biological questions. Metabolomics is an analytical tool to investigate small molecules in biological samples and can be used to investigate changes in several matrices. Metabolomics of mammalian cell culture models is now a promising tool with potential applications in many research areas. Reproducibility, repeatability and robustness of experimental methods are fundamental when performing metabolomics experiments to ensure results are reliable and contain biological insight. Numerous analytical techniques have been developed to measure the metabolome^1^, of which all have strengths and weaknesses. Mass spectrometry is one of the most common techniques used for metabolomics experiments and is very sensitive, but not inherently quantitative. Mass spectrometry can be used in isolation, by infusing a sample direct into the mass spectrometer (flow injection analysis or FIA) or it can be combined with a separation method such as liquid chromatography (LC). This increases the analysis time, but enables an extra measurement dimension which can assist with final identification and separation of individual compounds.

Metabolomic approaches have been applied to analyze a variety of mammalian cells, including primary cells, non-tumorigenic and tumorigenic cell lines as well as induced pluripotent stem cells. Compared to the analysis of body fluids, metabolomics analysis of mammalian cells involves additional sample processing steps, including quenching and metabolite extraction. There are many studies focusing on optimizing sample preparation and analytical methods for cell metabolomics and metabolic fingerprinting, although they are often very cell-type dependent^2–10^, primarily because the heterogeneity of different cell types makes a single optimal method difficult to achieve. Standardization is important within an experiment since many factors will affect the final results including culture conditions, cell density, number of cells analyzed and culture media composition.

The Absolute*IDQ* p400 HR kit from Biocrates Life Science AG is a commercial product for targeted metabolomics. The assay uses a combination of ultra-high-performance liquid chromatography-mass spectrometry (LC–MS) and FIA mass spectrometry methods for analysis. The kit covers 408 metabolites from 11 metabolite classes (amino acids, biogenic amines, hexose, acylcarnitines, diglycerides, triglycerides, lysophosphatidylcholines, sphingomyelins, ceramides cholesteryl esters and phosphatidylcholines) which can be simultaneously identified and quantified. The measured metabolites cover a broad range of metabolic pathways including those involved in inflammation, oxidative stress, fatty acid oxidation, insulin resistance, neurodegeneration, and signal transduction. The kit is often more expensive per sample than a home-developed method. However, the kit is designed to give broad overview of combined polar and non-polar (lipid) compounds in a standardized way that enables individuals with only medium mass spectrometry instrumentation knowledge to analyze the kits without much extra training. Entire individual pathways like glycolysis or tricarboxylic acid compounds are not covered by the Absolute*IDQ* p400 HR kit. While the Absolute*IDQ* p400 HR kit has been validated for human plasma and serum, adherent cell lysates have not yet been evaluated. Since matrix effects and concentration will affect the performance of mass spectrometry methods, a method validated for one matrix cannot be assumed to be valid for another matrix. The ability to use a kit-based method for cells is unlikely to be superior to existing methods which have been designed specifically for cell matrices, especially in terms of their sensitivity. However, it does allow both a broad coverage of metabolites, and its preparative and analytical methods are relatively fast and suitable for use by individuals less experienced in mass spectrometry.

We tested the analytical performance of the Absolute*IDQ* p400 HR kit on lysates prepared from an adherent osteosarcoma cell line. We present a cell preparation and analytical method and report on the performance of the kit with regard to detectability of the targeted metabolites and their repeatability.

## Results

In this study we tested extraction solvent type, cell mass used for extraction and loading volumes for adherent human osteosarcoma cells (HOS) using the Absolute*IDQ* p400 HR kit. Five biological replicates were analysed for each tested condition. Data processing was done with the Met*IDQ* software from Biocrates.

### Limitations of the kit for metabolite quantification

According to the Biocrates validation criteria, most metabolites were rejected due to being discoverable but below the lower limit of quantification (LLOQ) i.e. most of the values for the compounds in the samples were below the lowest calibration point provided in the kit but were visible in the mass spectrum. Therefore, new data processing steps were generated (see “Materials and methods” section).

Since we were expecting low concentrations of metabolites in our cell lysate extracts, many of which were below the stated level of quantification, we wished to determine if they could nevertheless be relatively quantified. We therefore tested this by using the “Cal 1” (calibration 1) solution that is provided in the Biocrates kit. This contains standards only for LC compounds but these are at known concentrations. This approach therefore enabled us to determine where the limit of detection and the limit of relative quantification was for our instrument for LC compounds. Cal 1 was serially diluted in water (1:1.2, 1:1.4, 1:1.6, 1:1.8, 1:2, 1:3, 1:5, 1:7.5, 1:10, 1:15, 1:20, 1:30, 1:50 and 1:100). Concentrations were specifically chosen to be within the limit of detection (LOD) and LOQ as determined by Biocrates for individual metabolites (Suppl. Table 1). These concentration curves were analyzed three times for each concentration and the relative concentration and the repeatability determined for each metabolite. The aim was not that each concentration would sit on a linear scale, but that, for any individual metabolite, an increasing concentration of that metabolite would be reflected by an increased mass spectrometric intensity. Suppl. Figure 1 shows the metabolites which were within the Biocrates calibration range and/or the compounds which were still in a linear range but below Biocrates LOD with our instruments using the HOS cell measurement. The metabolites Ac-Orn, alpha-AAA, carnosine, nitro-Tyr and spermine were excluded from further data analysis since they were below the LOD using our instruments in the HOS cells. Since the FIA part only contains 1-point calibration we were not able to verify the approach for these compounds.

### Determination of cell mass and loading volume

Cell count has traditionally been used as a normalization strategy for in vitro cultures. Typically, trypsination is used to release cells for easy counting. It is known that trypsination leads to alterations in the metabolism of cells and induces a lysis of lipid vesicles^11^. Alternatively, adjusting for cell mass could be used prior to chemical analysis as a normalization strategy. The linear relationship between cell mass and cell count in HOS cells is shown in Suppl. Figure 2. For HOS cells, we tested three different cell masses (5 mg, 15 mg and 25 mg) and two different lysate loading volumes (10 µL and 20 µL). Biocrates had made no recommendations for adherent cells at the time of writing, but had suggested using ethanol-phosphate (EtOH-P, 85/15 v/v) for suspension cells. Therefore, this extraction solvent was included for testing in our study on adherent cell lysates.

In the liquid chromatography (LC) measurement the highest number of amino acids and biogenic amines (28/42) were obtained using a cell mass of 25 mg and a loading volume of 20 µL (Table 1). However, more than 50% of the compounds included in the kit for LC measurement were detected in all experimental conditions studied. Citrulline was not detected at all, while lysine, methionine and tryptophan were present only in single conditions (lysine 15 mg/20 µL, methionine 15 mg/10 µL and 15 mg/20 µL, tryptophan 15 mg/10 µL, 5 mg/20 µL and 15 mg/20 µL. For biogenic amines only a set of 7/21 could be detected. Creatinine was only detected using 5 mg/20 µL and Met-SO could be only shown when using 15 mg/20 µL. The differences seen in measured areas and relative standard deviations (RSD) are discussed below. When a loading volume of 20 µL was employed, the FIA measurement revealed 158/366 (43%) and 153/366 (42%) detected compounds for 15 mg and 25 mg of cell mass, respectively. For the other conditions, less than 40% of the analytes could be measured. Monosaccharides could be detected only when the 25 mg cell mass was combined with 20 µL of loading volume, although this was at the expense of triglyceride detection. A Kruskall-Wallis test (p = 6.7 × 10^−4^ for LC and p = 3.9 × 10^−4^ for FIA) followed by a Tukey Kramer multiple comparison test was conducted to assess the significance of these differences by ranking the number of compounds from lowest (Rank 1) to highest (Rank 6) for each metabolite across the conditions (Suppl. Table 2). Missing values were ignored. Compared to the top performing 25 mg/20 µL condition, the 5 mg/20 µL and 5 mg/10 µL conditions were significantly different for the LC measurement. For the top performing condition (15 mg/20 µL) for the FIA measured compounds, significant differences were seen in the 5 mg/20 µL and 25 mg/10 µL conditions by Tukey Kramer testing. The significance levels quoted should be seen as conservative estimates of difference as significant differences were markedly increased in some tests if missing values were replaced with a level 6 category ranking, emphasizing that how missing values are treated can markedly affect interpretation of results.

**Table 1:** Number of detected compounds for different cell masses (mg) and loading volumes (µL) for both liquid chromatography (LC) and flow injection analysis (FIA) mass spectrometry using the Biocrates p400 HR kit.

Group	Measurement type	Compounds in total	Cell mass [mg]					
			5	15	25	5	15	25
			Loading volume [µL]					
			10	10	10	20	20	20
Amino acids	LC	21	16	18	19	16	18	19
Biogenic amines	LC	21	5	6	7	6	7	9
**Sum**		42	21	23	26	22	25	28
Hexose	FIA	1	0	0	0	0	0	1
Acylcarnitines	FIA	55	2	3	3	2	3	3
Diclycerides	FIA	18	3	4	4	4	5	5
Triglycerides	FIA	42	5	4	1	5	7	0
Lysophosphatidylcholines	FIA	24	5	5	4	4	5	8
Phosphatidylcholines	FIA	172	102	116	97	96	120	119
Sphingomyelins	FIA	31	14	16	9	10	16	15
Ceramides	FIA	9	1	0	0	1	0	0
Cholesteryl Esters	FIA	14	3	2	3	3	2	2
**Sum**		366	135	150	121	125	158	153
**Total**		408	156	173	146	147	183	179

Five replicates were measured in each condition; outliers were removed from the final analysis as described in the materials and methods. BR: Biological replicates. A strict noise threshold filter was applied to data such that in some cases, compounds were detected but filtered out before these results were compiled.

Significant values are in bold.

Relative standard deviation (RSD) is a measure of the repeatability of a result. The suggested maximum acceptance tolerance of the RSD in LC–MS metabolomics is 15% for any individual compound (https://www.ema.europa.eu/en/bioanalytical-method-validation).

**Figure 1:**
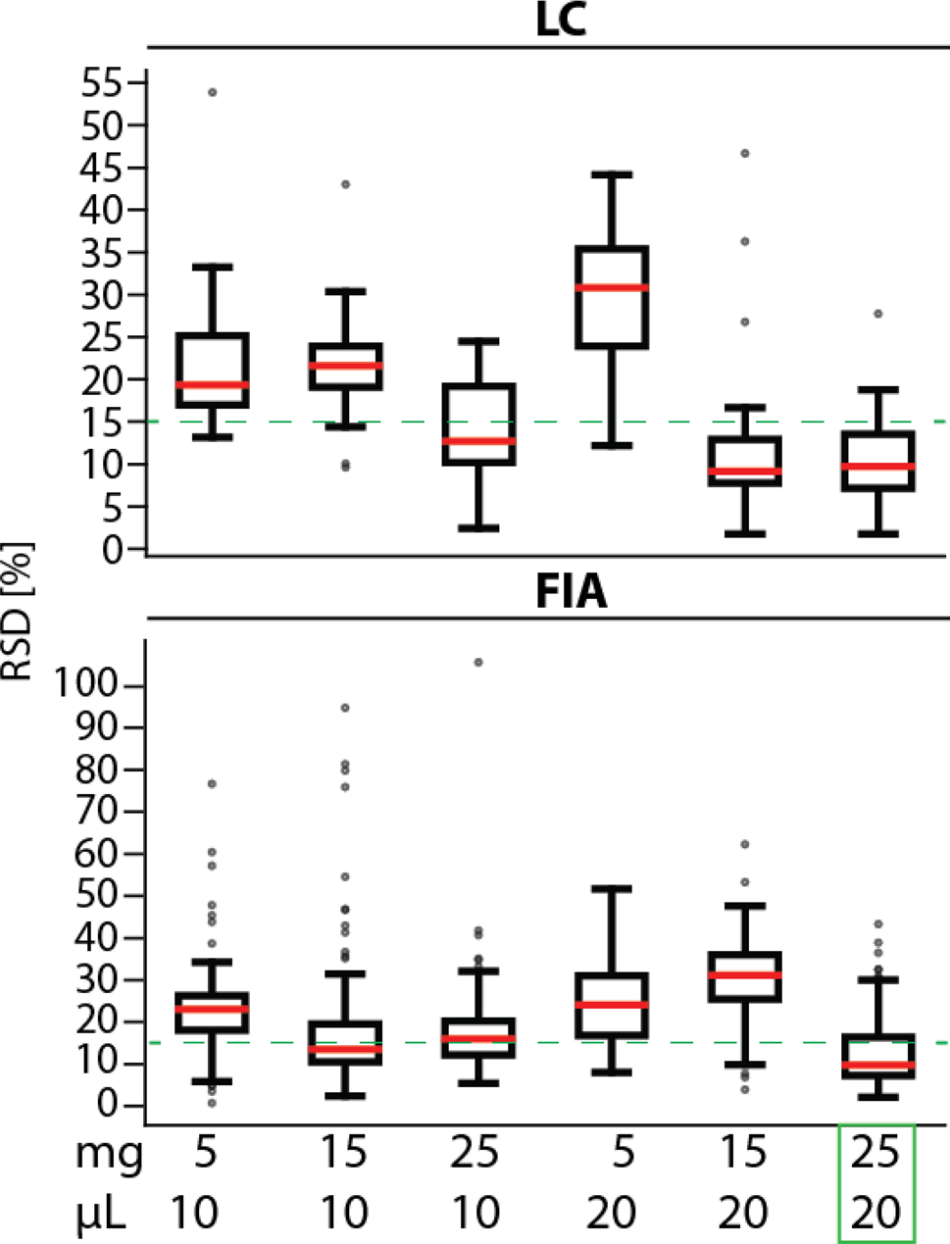
Boxplots of individual compounds’ relative standard deviation (RSD) using different cell masses (mg) and loading volumes (µL). Dashed line represents the quality assurance threshold of 15%. Five replicates were measured in each condition; outliers were removed from the final analysis as described in the materials and methods. Highlighted in green is the combination we consider the best. LC: liquid chromatography. FIA: flow injection analysis.

The individual compound RSDs are shown in Suppl. Table 3. For the LC–MS measurement, the lowest median RSD (9%) was found when 15 mg cell mass was extracted and a 20 µL loading volume was used (Table 2). Using the 25 mg cell mass gave very similar median RSDs (13% and 10%, respectively) for both 10 µL and 20 µL. A Kruskall-Wallis test (p = 1.2 × 10^−13^ LC and 1.1 × 10^−48^ FIA) followed by a Tukey Kramer test was conducted to assess the significance of these differences using a similar ranking system as described above. The 15 mg/20 µL condition had significantly different RSDs to all of the 10 µL loading conditions but not to the 20 µL loading conditions. For FIA measurement, two conditions shared the lowest median RSD of 13% (25 mg/20 µL and 15 mg/10 µL). In the FIA measurement the highest number of metabolites could be detected using 15 mg/20 µL; however, the median RSD and the percent of compounds below the threshold of 15% RSD was better in the 25 mg/20 µL condition. All conditions except the 15 mg/10 µL had significantly different RSDs to the 25 mg/20 µL condition as assessed by a multi comparison test. In summary the best conditions for cell lysis using EtOH-P is a combination of 25 mg cell mass and 20 µL of loading volume since a low median RSD of 10% and 13% were found in LC and FIA measurement, respectively combined with a high proportion of RSDs below 15% (81% LC, 59% FIA). However, this benefit was slightly offset by a decrease in number of detected compounds in particular classes (Figs. 1, 4).

**Table 2:** The median relative standard deviation (RSD) of individual compounds in HOS cells and the percentage of detected compounds with RSD < 15% (in total and in % from detected number) as detected with the Biocrates p400 HR kit using either liquid chromatography (LC) for flow injection analysis (FIA) mass spectrometry.

Measurement type	Criteria	Cell mass [mg]					
		5	15	25	5	15	25
		Loading volume [µL]					
		10	10	10	20	20	20
LC	Median RSD [%]	19	22	13	25	9	10
	Number of compounds with RSD < 15%	4/20 (20%)	3/23 (13%)	16/26 (62%)	5/22 (23%)	21/25 (84%)	22/27 (81%)
FIA	Median RSD [%]	23	13	16	24	31	13
	Number of compounds with RSD < 15%	26/136 (20%)	92/150 (61%)	52/121 (44%)	21/125 (17%)	12/158 (8%)	91/153 (59%)

Five replicates were measured in each condition; outliers were removed from the final analysis as described in the materials and methods.

### Determination of cell lysis extraction solvents

**Figure 2:**
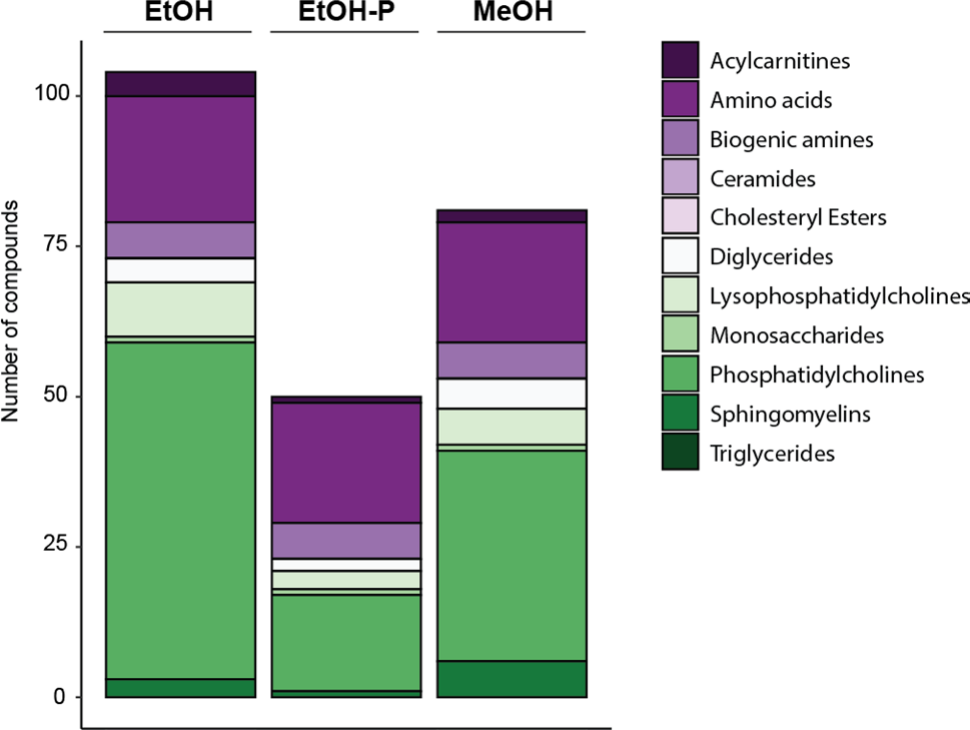
Number of detected compounds per biological group using different cell lysis solvents. Five replicates were measured in each condition; outliers were removed from the final analysis as described in the materials and methods. A strict noise threshold filter was applied to data such that in some cases, compounds were detected but filtered out before these results were compiled.

In the next step, we compared EtOH-P to pure ethanol (EtOH) and methanol (MeOH) for cell lysis in HOS cells. When comparing the three solvents, we found that EtOH (96/408) allowed the detection of a greater overall number of compounds compared to EtOH-P (49/408) or MeOH (80/408) (Fig. 2, Suppl. Table 4). These differences were principally due to the additional phosphatidylcholines detected. The number of amino acids and biogenic amines detected were broadly equivalent across the extraction solvents tested. Threonine was not detectable at all, while citrulline was detected only when EtOH was used as a lysis solvent.

**Figure 3:**
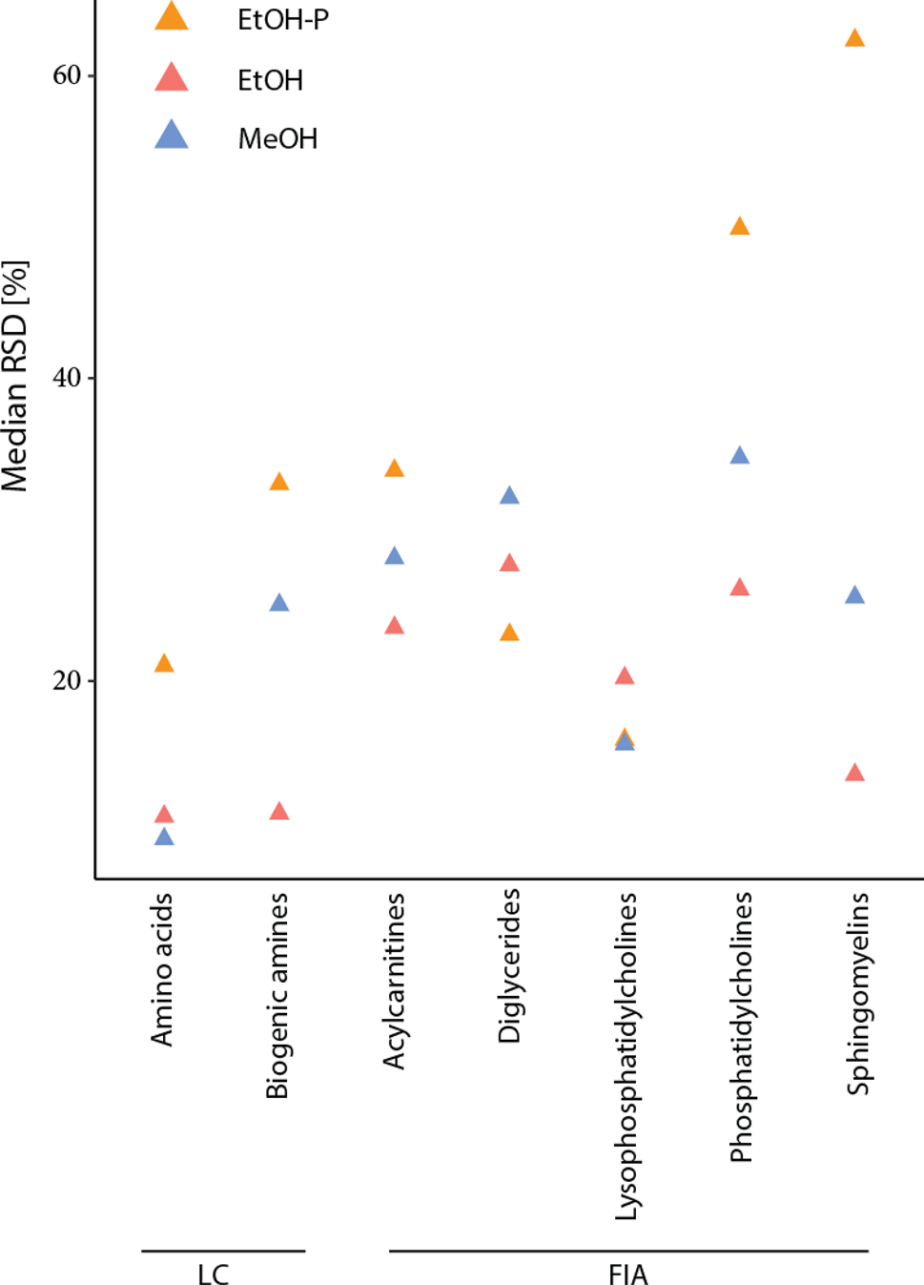
Results of an analysis of HOS cells using the Biocrates p400 HR kit using different cell lysis extraction solvents. Median relative standard deviation (RSD) of the individual compounds per biological group. Both liquid chromatography (LC) and flow injection analysis (FIA) mass spectrometry measurements are shown. Five replicates were measured in each condition; outliers were removed from the final analysis as described in the materials and methods.

For FIA measurement, EtOH performed better than EtOH-P as an extraction solvent, as measured by the total number of detected compounds (p = 7 × 10^−3^ by Kruskal Wallis, with p = 4.7 × 10^−3^ by Tukey Kramer, Suppl. Table 4). Ceramides and cholesteryl esters were detected when using 25 mg/20 µL with EtOH-P buffer in the first study (cell mass and loading test), while they were not detected in the second study (solvent test). For metabolites measured by LC, median RSDs were low for EtOH (9%) and MeOH (11%) but higher for EtOH-P (25%). For FIA, the use of pure EtOH clearly improved the median RSD (23%) compared to EtOH-P (43%) or MeOH (30%) (Table 3, Fig. 3). A significant difference between the RSDs using a Kruskal–Wallis Tukey Kramer test was shown for EtOH and MeOH compared to EtOH-P for LC (Kruskal Wallis p value = 5.2 × 10^−9^) and FIA (Kruskal Wallis p value = 2.3 × 10^−8^) measurements. For both LC and FIA measurement types, the highest number of metabolites detected overall (77) and the highest number detected with an RSD < 15% threshold was shown for cell lysis using EtOH. The difference in the number of detected metabolites was only significant when compared to EtOH-P (p = 0.04 and 5 × 10^−3^ for LC and FIA respectively, Tukey Kramer result of EtOH-P vesus EtOH). The comparison of the median RSDs per biological group revealed similar results. Median RSDs were good for both pure solvents for the amino acids (10% and 11% for EtOH and MeOH respectively), but was considerably higher for EtOH-P (21%). For biogenic amines and amino acids, the use of EtOH and MeOH reached RSDs at about 15% threshold for the majority of the metabolites. Acylcarnitines, diglycerides and phosphatidylcholines showed high RSDs independent of the buffers used. For sphingomyelins the most reproducible results were achieved when EtOH was used (median RSD of 14%), although more were actually detected when MeOH was employed (6 out of a possible maximum of 8 for MeOH, compared to 3 for EtOH and 1 for EtOH-P). The individual metabolite RSDs are shown in Suppl. Table 6.

**Table 3:** The median relative standard deviation (RSD) of the individual compounds in HOS cells and the percentage of compounds with a RSD < 15% threshold (in total and in % from detected number) for liquid chromatography (LC) and flow injection analysis (FIA) mass spectrometry.

Measure type	Lysis buffer	EtOH-P	EtOH	MeOH
LC	Median RSD [%]	25	9	11
	Compounds with RSD < 15% [%]	3/26 (12%)	23/27 (85%)	23/26 (88%)
FIA	Median RSD [%]	43	23	30
	Compounds with RSD < 15% [%]	3/24 (13%)	16/77 (21%)	4/55 (7%)

Five replicates were measured in each condition; outliers were removed from the final analysis as described in the materials and methods.

To test whether the results are comparable in other cell lines, the MNNG/HOS and 143B osteosarcoma cell lines were extracted using the same solvents (EtOH-P, EtOH, MeOH). A similar number of detected compounds could be shown for the LC measurement (a median of 27 and 28 compounds for EtOH extraction respectively). For MNNG/HOS cells, this was only significantly different between EtOH phosphate (25 compounds) and MeOH (29 compounds) with a Turkey Kramer p value = 2.5 × 10^−3^. These numbers represent coverage of 59% (EtOH-P) 64% (EtOH) and 69% (MeOH) of all possible LC compounds. The corresponding percentages for 143B cells were 67% (EtOH-P), 67% (EtOH) and 59% (MeOH) respectively (Suppl. Table 7). For the FIA measurement the highest number of metabolites could be detected using EtOH as lysis buffer (32% and 36% in MNNG/HOS and 143B cells, respectively). This was considerably better than MeOH (Turkey Kramer p value = 0.048) for both cell types, and for EtOH-P for the 143B cells (Turkey Kramer p value = 5.9 × 10^−3^) The MNNG/HOS cells showed the lowest median RSD using EtOH extraction solvent in the LC measurement (18% EtOH-P, 8% EtOH and 21% MeOH); in the FIA measurement the lowest median RSD by some margin was again with EtOH (15%). For 143B cells a lower median RSD could be shown when cells were lysed in EtOH solvent for FIA measurement (23%) compared to EtOH-P (62%) or MeOH (71%). For the 143B cells, both number of compounds and RSD results are likely strongly influenced by overall poorer sensitivity seen in this cell culture (including in proteomics studies not covered in this paper). For LC measurement a lower median RSD was shown using MeOH (14%) compared to the other tested extraction solvent (24% EtOH-P, 21% EtOH).

**Figure 4:**
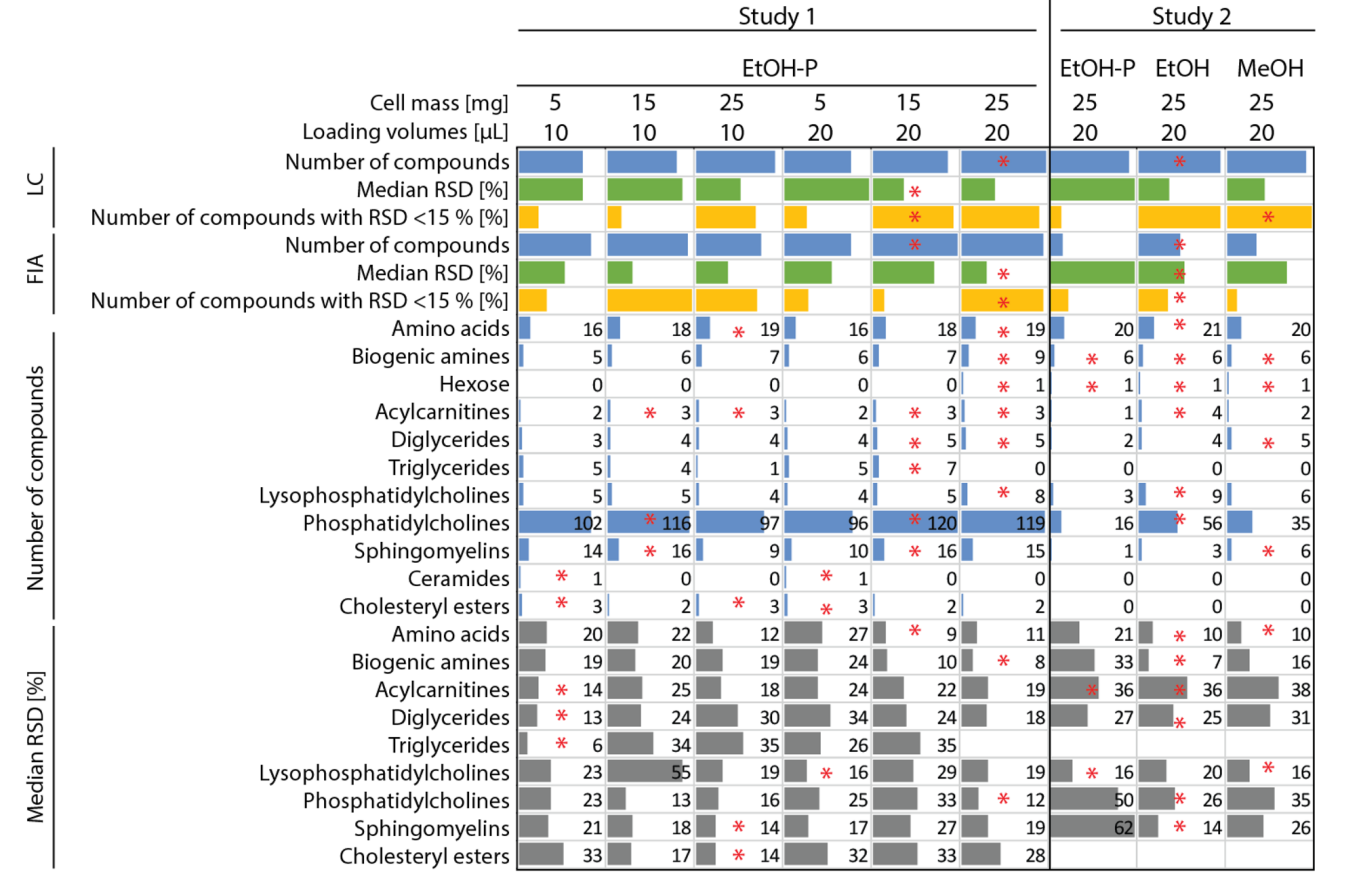
Summary of the obtained results from both the cell and volume determination (study 1) and the extraction solvent determination (study 2) in HOS cells. Red stars indicate the best condition either in study 1 or study 2 for the analyzed objects. LC: liquid chromatography. FIA: flow injection analysis.

In summary, EtOH appears to be the better solvent to conduct cell lysis (Fig. 4). When detection of the lipid species is a priority, consideration should be given to including a more organic solvent in the cell lysis buffer. For polar metabolites the three buffers revealed very similar results with regard to detectability and repeatability. Further studies are required to assess batch to batch validation and the analysis of other cell types and conditions.

## Discussion

The Absolute*IDQ* p400 HR kit has been previously validated for the analysis of human serum and plasma^12^. In this work, we set out to optimize the detection of polar and lipid metabolites using the Absolute*IDQ* p400 HR kit to enable repeatible and robust analysis. We focused on optimizing the starting total cell mass, the extraction solvent use, and the volume loaded onto the kit plate. Since the metabolite concentrations were below the smallest calibration standard provided in the kit, a relative analysis was done i.e. sample intensities were assessed relative to the intensities detected in other samples.

As we reached the maximum confluency of a standard cell culture dish, the usage of 25 mg per sample is the maximum possible value available for this type of cell culture. Increasing sample amounts by pooling multiple cultures or similar measures are not normally practical, especially where experimental designs already necessitate large numbers of culture plates. We explored why the results between the first (cell mass and loading volume determination in HOS cells) and the second study (different extraction solvents used in HOS cells) were so different. Comparing results from the first and second studies of 25 mg/20 µL extracted in EtOH-P in HOS cells, we observed that the number of compounds detected were substantially different. This could be due to an overall decrease in detected intensity in the second study, possibly due to a lower starting number of cells extracted, or to a slight drop in sensitivity of the instrument. This led to many compounds which were included in the first study being filtered out in the second by a strict noise threshold of 20,000 counts (as recommended by Biocrates). A system suitability test was run before each study suggesting that the instrument was performing to specification each time, but this still enables a degree of sensitivity drop as the instrument is used over time and becomes dirtier.

The metabolite panel in the kit includes metabolites with different lipophilic and hydrophilic properties. Therefore, the most commonly used extraction solvents recommended by the manufacturer were tested (EtOH-P, EtOH and MeOH). Biphasic extractions split the sample into polar and non-polar components and therefore are more difficult to use with such a kit. We found that the extraction solvent used has a clear impact on the number of metabolites being retained and the repeatability of the replicates. Since we used the Biocrates recommended noise threshold of 20,000 counts, metabolites were not retained in conditions where they failed to reach this intensity. This explains some of the wide variation seen. As anticipated, the impact was metabolite class dependent. Biogenic amine, acylcarnitines, phosphatidylcholines and sphingomyelins could be detected with a better repeatability when using EtOH instead of EtOH-P or MeOH. In contrast, diglycerides showed a better repeatability when extracted in EtOH-P compared to EtOH or MeOH. Phosphatidylcholines were detected in both EtOH and MeOH at approximately the same rate, but were slightly more intense in EtOH extracted samples (median intensity 11,817 for MeOH and 42,713 in EtOH). By contrast, the intensity of sphingomyelins was higher in MeOH extracted cells (median intensity 25,785 for MeOH and 15,058 in EtOH). MeOH also performed best for triglycerides in the 25 mg/20 µL loading condition. Overall, an extraction solvent of pure EtOH resulted in the highest number of detected and retained metabolites with good metrics of repeatability when using the Biocrates Absolute*IDQ* p400 HR Kit. This probably reflects the relatively large proportion of lipids in the kit, and the relatively less polar nature of ethanol compared to the other solvents employed. It is not clear whether the deleterious effect of additional phosphate buffer was mostly due to the chemistry of extraction, or the phosphate causing ion suppression for some species^13^.

The limits of our optimization are that for practical reasons, we were unable to carry out a full validation as recommended by the FDA or EMA. In part this was due to the proprietary nature of the kit meaning we did not know the original starting concentrations of the internal standards used for the FIA. We were limited in the number of cell lines available to us for testing. As only three cell lines have been tried using this method it cannot be assumed that our results for these cell types are generalizable to other cell lines, especially from other species (particularly bacterial or plant species where the cell walls may require a different approach). As mentioned above, average cell size and number also differ between cell types and have an influence on the cell mass available and/or required for measurement. This may also differ between tumor cell lines. For example a tested acute amyloid suspension cell line was measured using 5 mg of cell pellet, although this also revealed EtOH as the best solvent for extraction of those tested (unpublished results).

The previous version of the Absolute*IDQ* p400 HR kit is the Absolute*IDQ* p180 kit from Biocrates. The Absolute*IDQ* p400 HR kit covers all compounds from the p180 kit with an extension of acylcarnitines (n = 15 more), glycerophospholipides (n = 106 more) and sphingomyelins (n = 16 more) and an addition of ceramides, cholesteryl esters, di- and triglycerides. Biocrates have produced an application note (Biocrates application note 2001–1) for the use of this kit with cell lysates from suspension cells. Crucially, the p180 kit was designed for use with triple quadrupole and Qtrap instruments and so is not directly comparable with the p400 HR kit which was specifically developed for orbitrap systems. The p180 kit covers a much reduced number of metabolites compared with the p400 HR kit and the application note compared only EtOH-P and PBS as lysing solvents. However, results were broadly comparable with what we have shown with the p400 HR kit. Namely, the overall number of detected compounds was similar but PBS was the preferred solvent for amino acid and biogenic amine extraction and EtOH-P performed better on lipid species. We did not compare PBS alone, but these results only partially confirm ours. We did indeed see that a higher organic solvent ratio improved the detection of lipids (in this case, EtOH outperformed both EtOH-P and MeOH). Perhaps surprisingly, EtOH also performed best for amino acids and biogenic amines. We would not have predicted this, but could hypothesize that the use of 100% alcohol is more efficient at denaturating proteins and preventing *ex-vivo* metabolism^14^. In the only direct comparison we have between the two kits, we can see that fewer compounds were detected overall in our method compared to the application note (50 versus 120) when EtOH-P was used. We assume this is due to a lower number of starting cells to extract (4 × 10^+7^ cells/mL and 6 × 10^+6^ cells/mL for the application note and our study, respectively). When using the p400 HR kit with human plasma (pooled plasma NIST SRM-1950), 222 metabolites were routinely quantified using orbitrap instruments shown by an international ring trial^15^. However, with routine use of the kit, plasma samples are not first extracted using alcoholic solvents before measurement. As anticipated, the metabolites seen in cell cultures were also different compared to plasma, but were perhaps more similar for polar metabolites than anticipated. In the ring trial, 21 amino acids and 8 biogenic amines could be detected compared to 21 amino acids and 6 biogenic amines in our HOS cell culture when EtOH was used as a lysis solvent. Regarding the FIA measurements, our study of cells detected more glycerophospholipids compared to the ring trial (our study 112, ring trial 79). As glycerophospholipids form a major part of cell membranes, this result is plausible. For other lipid groups fewer compounds were measured in our HOS cells than in the ring-trial-plasma (acylcarnitines 25 and 47, cholesteryl esters 4 and 11, glycerolipids 27 and 47 and sphingomyelins 16 and 27 for our study and ring trial, respectively). Blood is a major transporter for many of these lipids. There may also be matrix effects at play in the mass spectrometric analysis which would speak against directly comparing results from different matrices based on intensity readings alone.

We also observed differences in the metabolite coverage between the first and second study we analyzed. Ceramides and cholesteryl esters were detectable in the first, but not the second study even when the same extraction solvent was used. However, the absolute number of compounds this applied to was low (3/9 ceramides and 3/14 cholesteryl esters). The same was found for triglycerides from the FIA measurement. The raw data showed that species from these lipid classes were close to the cut off threshold of 20,000 counts in both studies with them being just above the threshold in the 1st study and slightly below in the 2nd study. This clearly demonstrates the oft-cited mass spectrometry adage that a missing value does not mean a zero value. We hypothesize that this problem would be less common, and the data between studies more reproducible, if we were to use a higher starting mass of cells. Since this option is not always available in cell culture studies, it may be worth addressing in future studies whether the absolute threshold recommended by Biocrates can be reliably reduced for cell cultures.

Lipids are a heterogeneous class of organic substances with different hydrophilic tendencies. Using larger percentages of organic solvent (100% EtOH or 100% MeOH) led to increased detectability of phosphatidylcholines and lysophosphatidylcholines compared to EtOH-P probably due to their physicochemical properties of solvation preferring a less hydrophilic environment. In comparison, amino acids and biogenic amines are more hydrophilic and the number of detected compounds in these classes was not substantially different across the three solvents tested. However, the median RSD values revealed a better reproducibility with pure alcohol as an extraction solvent.

The results presented in this study reveal that the Absolute*IDQ* p400 HR Kit can be used for analyzing cell lysates from adherent osteosarcoma cell lines. We assume that the procedure can be adapted for other adherent cells cultivated under similar conditions; however, only a relative data analysis is possible using the kit for measurement of human adherent cells. We believe however that absolute quantification, while useful, is of less importance to most scientists analyzing cell cultures. The measurement of certain compounds should be considered unreliable and this is of scientific importance to cell researchers. We would also recommend running a diluted calibration curve in each batch (in addition to the standard calibration range recommended by Biocrates) to ensure that results are within a reliable relative quantification range. Quality assurance measures should also be applied to report only between-group differences which exceed appropriate limits of technical variation. Number and characteristics of outliers can inform on the performance of the method. Since the analysis of metabolic compounds is influenced by many factors we strongly recommend the inclusion of a pilot study when starting with new cell lines to determine the most appropriate experimental conditions. Dependent on the quality of the measurement up to 45% of the metabolites from the Absolute*IDQ* p400 kit can be detected with a good analytical performance. We emphasize that these results were based on our instrument and different instruments, even of the same model, may have different sensitivity thresholds. The kits are relatively expensive and their principle advantage may be to support users with less analytical method development experience and without full bioinformatics support.

The ability to use a kit-based method for cells is unlikely to be superior to existing methods designed specifically for cell matrices, especially in terms of their sensitivity, but it does allow both a broad coverage of metabolites, and a faster and more flexible method to be used, including by individuals less expert in mass spectrometry. We recommend understanding the limitations of using these kits for cell analysis, but can broadly recommend that they can be used for this purpose with appropriate caveats. We recommend using EtOH as a broad-based extraction solvent for cell lysates and 25 mg of cell mass with a plate loading volume of 20 µL per sample when using the Biocrates Absolute*IDQ* p400 HR Kit. This combination allows analysis of a broad lipid and metabolic profile of various compound classes for the cell types we tested.

## Materials and methods

### Cell culture

The cell lines HOS and MNNG/HOS were obtained from ATCC (American Type Culture Collection, Teddington, United Kingdom). The 143B cell line was purchased from Sigma-Aldrich (St. Louis, Missouri, US). All cells were grown at 37 °C and 5% CO_2_ in RPMI 1640 media (GIBCO, Carlsbad, CA, USA) supplemented with 10% fetal bovine serum (GIBCO) and 1% penicillin/streptomycin (Gibco). Cells were plated in a 10 cm^2^ cell culture dish with an approximate confluency of 70–90% on the day of harvest. Five biological replicates were grown for each tested condition.

### Determination of cell mass and loading volume

#### Cell mass determination

After removal of the culture medium, cells were washed with pre-cooled (4 °C) 0.9% sodium chloride solution (Sigma-Aldrich). After washing, 1.5 mL of 0.9% sodium chloride was added to the tissue culture dish and the cells were quickly harvested using a cell scraper and transferred into pre-weighed vials (1.5 mL Eppendorf tube). Cells were centrifuged at 4 °C for 3 min at 452×*g*. Afterwards, the supernatant was carefully removed and the wet pellet was weighed. After weighing, the pellets were quickly snap frozen in liquid nitrogen and stored at −80 °C until lysis.

#### Determining optimum cell mass to extraction solvent ratio

For repeatable mass spectrometry measurements, the material needs to be concentrated enough that analytes are reliably detected and the solvent volume needs to be sufficient to extract the cell pellet in a repeatable way. Additionally, the Biocrates kit has a loading volume limit of 20 µL per well per drying cycle. If the user wishes to load more than this amount, a drying step is required in-between. Loading more than 20 µL/well thus increases the preparation time and the number of steps required, and thus the potential for experimental error. We therefore limited ourselves to testing amounts that were achievable from a single sample plate harvest, and with loading volumes that required only a single step. To assess this, the frozen pellets were thawed on ice and suspended in ethanol-phosphate buffer (EtOH-P, v/v 85/15; EtOH and 1 × phosphate buffered saline (1 tablet/200 mL H_2_O equal to 0.01 M phosphate buffer, 0.00278 M potassium chloride and 0.137 M sodium chloride) (Sigma-Aldrich) in a volume of 25 µL/5 mg, 25 µL/15 mg or 25 µL/25 mg cell mass. The samples were sonicated for 3 min and snap frozen in liquid nitrogen for 30 s. The sonication and snap freezing steps were repeated twice. Afterwards the lysates were centrifuged for 5 min at 4 °C at 18,213xg. The supernatant was collected into a new vial (1.5 mL Eppendorf tube) and stored at −80 °C until use. All samples for this experiment were measured as a single batch on a single Biocrates p400 HR plate.

### Determination of cell extraction solvent

Cell harvest and cell lysis was performed as described in the previous section. The frozen pellets were thawed on ice and suspended in EtOH-P, EtOH or MeOH with 25 µL/25 mg. EtOH-P was used as it had previously been recommended by Biocrates for cell cultures. EtOH was tested to determine how phosphate buffer influenced the extraction. MeOH was used as a more polar comparison, and because it traditionally performs better under some LC–MS conditions due to its lower viscosity. All three solvents have been previously recommended by Biocrates for use with their kits. Samples were analyzed as a single batch on a single plate, on a different day compared to the optimal cell mass experiment.

### AbsoluteIDQ p400 HR assay and sample preparation

All reagents, internal and calibration standards, quality controls, and a patented 96-well filter plate required for Absolute*IDQ* p400 HR analysis were included in the kit provided by Biocrates Life Science AG (Innsbruck, Austria).

The Absolute*IDQ* p400 kit from Biocrates Life Science AG is a kit based on phenylisothiocyanate (PITC) derivatization of the target analytes using internal standards for quantification for all compounds. Amino acids (21) and biogenic amines (21) are determined using liquid chromatography mass spectrometry (LC–MS). Hexoses (1 compound which is a combined result for all hexoses of which glucose is normally by far the most prominent), neutral lipids like acylcarnitines (55), cholesteryl esters (14), diglycerides (18) and triclycerides (42) as well as polar lipids such as phosphatidylcholines (172), lysophosphatidylcholines (24), sphingomyelins (31) and ceramides (9) are analyzed by flow injection analysis (FIA). Cell sample preparation was carried out according to the manufacturer’s protocol.

Briefly, 10 µL or 20 µL of lysed cells and 10 µL internal standard were transferred to the upper 96-well plate and dried under a nitrogen stream. Thereafter, 50 µL of a 5% PITC solution was added to derivatize amino acids and biogenic amines. After incubation, the filter spots were dried. Metabolites were extracted using 5 mM ammonium acetate in MeOH (300 µL) into the lower 96-well plate for analysis after further dilution (150 µL extract plus 150 µL H_2_O for LC; 10 µL extract plus 490 µL FIA solvent for FIA). Quantification was carried out using internal standards (LC and FIA measurement) and a calibration curve (Cal 1 to Cal 7) for LC measurement only. The full list of metabolites and their abbreviations are listed in Suppl. Table 8.

Evaluation of the instrument performance prior to sample analysis was assessed by the system suitability test (SST) according to the manufacturer’s instructions (Suppl. Figure 3). Separate test mixtures were provided with the kit for LC–MS and FIA-MS SST evaluation. Pooled samples (pooled after extraction) were prepared at the same time as the cell samples and four to six pooled samples were used as an additional quality control throughout the run. The samples were randomized in each batch.

The LC–MS system was comprised of a 1290 Infinity UHPLC-system (Agilent, Santa Clara, CA, USA) coupled to a QExactive Plus (Thermo Fisher Scientific, Waltham, MA, USA) in electrospray ionization (HESI) mode. Calibration tune parameters were set to full MS mode with a scan range of 150.0 to 2,000 m/z with a resolution of 70,000 in positive mode with an AGC target 1 x 10^6^ and a maximum injection time of 50 ms. The sheet gas flow rate was in a range of 7–15 L/min, spray voltage 3.60 kV, capillary temperature 320 °C, S-lens RF level 60 and auxiliary gas heater temperature 30 °C with aux gas flow rate in a range of 1–3 L/min. Amino acids and biogenic amines were analyzed via LC–MS in positive mode with a runtime of 0 to 5.5 min, resolution of 70,000 or 35,000 for LC1 and LC2, respectively, AGC target 1 x 10^6^, 200 ms and a scan range of 55 to 800 m/z. Twenty microliters of the sample extract were used in the FIA in positive mode to measure acylcarnitines, glycerophospholipids, and sphingolipids, while hexose was monitored in a subsequent run in positive mode but with a different mass range (see below). The sheet gas flow rate was 60 L/min, spray voltage 3.0 kV, capillary temperature 300 °C, S-lens RF level 60 and 90 for LC1 and LC2, respectively and auxiliary gas heater temperature 550 °C with aux gas flow rate 30 L/min. Five µL of the diluted sample extracts were injected onto an Phenomenex 2.1 mm ID column (proprietary Thermo C18 column provided by Biocrates, length 10.5 cm) protected by an Phenomenex SecurityGuardTM ULTRA Cartridge C18/XB-C18 at 50 °C using a 6.81 min solvent gradient employing 0.2% formic acid in water (solvent A) and 0.2% formic acid in acetonitrile (solvent B). The prepared plate for LC was measured twice with different methods to obtain different compounds. The LC gradient for LC1 and LC2 method is shown in Suppl. Table 8. Twenty microliters of the diluted sample extract were used in the FIA in positive mode to capture acylcarnitines, glycerophospholipids, and sphingolipids, while hexose were monitored in a subsequent run in positive mode. All FIA injections were carried out using the Biocrates Running Solvent (290 mL MeOH plus 10 mL FIA reagent from the Biocrates kit). For FIA 1 a runtime of 0 to 3.01 min in positive mode with resolution of 70,000, AGC target 3e6, maximum IT 250 ms and 8 scan ranges (1: 150 to 170 m/z, 2: 170 to 200 m/z, 3: 200 to 240 m/z, 4: 240 to 256 m/z, 5: 390 to 520 m/z, 6: 520 to 634 m/z, 7: 634 to 730 m/z and 8: 730 to 931 m/z) were used. For FIA 2 a runtime of 0 to 3.01 min in positive mode with resolution of 70,000, AGC target 3e6, maximum IT 250 ms and 8 scan ranges (1: 256 to 280 m/z, 2: 280 to 305 m/z, 3: 305 to 335 m/z, 4: 353 to 363 m/z, 5: 363 to 390 m/z, 6: 390 to 415 m/z, 7: 415 to 445 m/z and 8: 445 to 570 m/z) was used. Settings were as follows: sheet gas flow rate was 15 L/min, spray voltage 2.5 kV, capillary temperature 300 °C, S-lens RF level 60 and auxiliary gas heater temperature 120 °C with aux gas flow rate of 5 L/min. The prepared plate for FIA was measured twice with different methods to obtain different compounds. FIA gradient for FIA1 and FIA2 method is shown in Suppl. Table 10. All compounds were identified and quantified using isotopically-labeled internal standards and multiple reaction monitoring (MRM) for LC and full MS for FIA as optimized and raw data was computed in Met*IDQ*™ (version 7.13.11-DB109-Nitrogen-2850) (Biocrates).

### Data analysis

The Met*IDQ* software is part of the Absolute*IDQ* p400 kit from Biocrates Life Science AG and is usually used for peak picking, identification and quantification. The concentrations of metabolites that were analyzed by FIA were automatically calculated by the software. The analyte peaks obtained by liquid chromatography (LC) were integrated by the Thermo Xcalibur software V4.3. The concentrations were determined based on the standards for calibration by applying the associated quantification method provided with the kit. With regards to the compound determination, we diluted Cal1 and added the data points as samples in MetIDQ i.e. they were treated as unknown samples. Following identification, we used our new data processing method and calculated the normalized peak area which we plotted in correlation to the theoretical concentration we expected for the different dilution points.

A modified version of MeTaQuaC (https://github.com/bihealth/MeTaQuaC) ^16^ was used for data quality analysis and filtering (language GNU R, version 0.1.31, R version 3.6.1). The modification enabled the retention of metabolites considered above the limit of detection but below the limit of quantification as defined by Biocrates software. In the adapted MeTaQuaC script, the status filters from the Biocrates MetIDQ software previously used to filter out compounds which were not quantifiable were ignored.

Metabolites were considered valid when they appeared in a minimum of 60% (normally three out of five) of biological replicates. Only analytes with values above the LOD were considered. The LOD for individual analytes was defined as three times the median peak area in the PBS samples (peak intensity was used for FIA data) and a minimum of 20,000 intensity in counts per second (cps). Analytes below the LOD were rejected. Each analyte was subsequently normalized to its respective labelled internal standard. For each study and condition n = 5 replicates were measured. Sample outlier detection was performed by calculating the median plus or minus 2.5 times the median absolute deviation from the normalized sum of area (LC) or intensity (FIA)^17^. Peak intensity was used for FIA data. A minimum of 4 out of 5 replicates were considered for analysis. Finally, compounds with less than 3 values per conditions and metabolite were discarded from the downstream analysis. The performance of the measurement was evaluated according to the following criteria: (1) number of detected metabolites, and (2) repeatability of the measurement of peak area or intensity. Statistics were performed using a non-parametric Kruskal–Wallis test on all groups followed by a Tukey Kramer test to confirm which groups were statistically significant. For an assessment of the overall repeatability of each experiment, for each metabolite, RSDs were ranked from 1 to n where n was the maximum number of conditions. A Kruskal Wallis was then performed on the ranks. This avoided metabolites with large RSDs from biasing the statistics. All statistics were performed in Matlab v R2018a with statistics toolbox. Missing values were denoted as NaN in the test and ignored for the purposes of testing statistical significance. Statistical significance was set at 95% (p ≤ 0.05).

## Supplementary Information

Supplementary Information.

## Data Availability

The datasets used and analyzed in the current study are available from the corresponding author on reasonable request. Normalized data for all the measurements made in the manuscript are provided as Excel file.
