# Supplementary material for: Analysis of adherent cell culture lysates with low metabolite concentrations using the Biocrates AbsoluteIDQ p400 HR kit
Source: Sci Rep. 2022 May 13;12:7933. doi: 10.1038/s41598-022-11118-7 (PMC9106690; doi:10.1038/s41598-022-11118-7)
Supplement: Supplementary file 1 — Supplementary Information. [file 41598_2022_11118_MOESM1_ESM.pdf]

Supplementary material for:

Analysis of adherent cell culture lysates with low metabolite concentrations using the Biocrates Absolute*IDQ* p400 HR kit

Raphaela Fritsche-Guenther[1], Yoann Gloaguen[1, 2], Alina Eisenberger[1] and Jennifer A. Kirwan[1, *]

[1] Berlin Institute of Health (BIH) @ Charité, BIH Metabolomics Platform, Lindenberger Weg 80, 13125 Berlin, Germany

[2] Berlin Institute of Health (BIH) @ Charité, Core Unit Bioinformatics, Chariteplatz 1, 10117, Berlin, Germany

*** Corresponding author:**

jennifer.kirwan@bih-charite.de

Supplementary Table 1. Calibration 1 (Cal 1) concentrations used for the dilution series of liquid chromatography (LC) compounds. Cal1 represent the lower limit of quantification (LLOQ).

**Compound**	**Cal1 [µM]**	**LOD**
Ala	20	9
Arg	5	2
Asn	5	1.4
Asp	5	1.5
Cit	5	0.1
Gln	20	3.7
Glu	10	3
Gly	25	4.7
His	5	1
Ile	5	1.5
Leu	5	1.6
Lys	10	1.4
Met	5	1.8
Orn	5	1.6
Phe	5	1.6
Pro	10	4
Ser	5	2
Thr	5	1.5
Trp	5	1.8
Tyr	5	1.7
Val	10	4.4
Ac-Orn	0.5	0.1
ADMA	0.25	0.1
SDMA	0.1	0.02
alpha-AAA	1	0.3
Carnosine	0.5	0.2
Creatinine	10	6
DOPA	0.5	0.2
Dopamine	1	0.7
Histamine	1	0.7
Kynurenine	1	0.5
Met-SO	1	0.06
Nitro-Tyr	1	0.3
PEA	0.1	0.001
Putrescine	0.1	0.07
Sarcosine	1	1
Serotonin	0.1	0.04
Spermidine	0.25	0.2
Spermine	0.25	0.2
Taurine	2.5	2.5
c4-OH-Pro	1	0.3
t4-OH-Pro	1	0.5

Supplementary Table 2: Tukey Kramer post hoc statistical results of number of compounds detected in HOS cells for liquid chromatography (LC) and flow injection analysis (FIA) parts of the Biocrates analysis comparing different loading amounts. Comparisons deemed significantly different are highlighted in bold. A Kruskal Wallis test was performed prior to the Tukey Kramer test. *CI = confidence interval. [&] Signifies 10 mg cell mass used, of which 10 µL was loaded onto Biocrates plate.

Type	Comparison 1	Comparison 2	Lower limit of CI*	Estimated difference between group means	Upper limit of CI*	Tukey Kramer p value
LC results	5 mg[&], 10µL[&]	15 mg, 10µL	-22.60	-9.00	4.60	0.41
	5 mg, 10 µL	25 mg, 10 µL	-30.38	-15.95	-1.52	0.02
	5 mg, 10 µL	5 mg, 20 µL	-17.88	-3.45	10.98	0.98
	5 mg, 10 µL	15 mg, 20 µL	-27.13	-12.70	1.73	0.12
	5 mg, 10 µL	25 mg, 20 µL	-34.13	-19.70	-5.27	0.00
	15 mg, 10 µL	25 mg, 10 µL	-21.38	-6.95	7.48	0.74
	15 mg, 10 µL	5 mg, 20 µL	-8.88	5.55	19.98	0.88
	15 mg, 10 µL	15 mg, 20 µL	-18.13	-3.70	10.73	0.98
	15 mg, 10 µL	25 mg, 20 µL	-25.13	-10.70	3.73	0.28
	25 mg, 10 µL	5 mg, 20 µL	-2.71	12.50	27.71	0.18
	25 mg, 10 µL	15 mg, 20 µL	-11.96	3.25	18.46	0.99
	25 mg, 10 µL	25 mg, 20 µL	-18.96	-3.75	11.46	0.98
	5 mg, 20 µL	15 mg, 20 µL	-24.46	-9.25	5.96	0.51
	5 mg, 20 µL	25 mg, 20 µL	-31.46	-16.25	-1.04	0.03
	15 mg, 20 µL	25 mg, 20 µL	-22.21	-7.00	8.21	0.78
FIA	5 mg, 10 µL	15 mg, 10 µL	-22.60	-9.00	4.60	0.41
	5 mg, 10 µL	25 mg, 10 µL	-30.38	-15.95	-1.52	0.02
	5 mg, 10 µL	5 mg, 20 µL	-17.88	-3.45	10.98	0.98
	5 mg, 10 µL	15 mg, 20 µL	-27.13	-12.70	1.73	0.12
	5 mg, 10 µL	25 mg, 20 µL	-34.13	-19.70	-5.27	0.00
	15 mg, 10 µL	25 mg, 10 µL	-21.38	-6.95	7.48	0.74

	15 mg, 10 µL	5 mg, 20 µL	-8.88	5.55	19.98	0.88
	15 mg, 10 µL	15 mg, 20 µL	-18.13	-3.70	10.73	0.98
	15 mg, 10 µL	25 mg, 20 µL	-25.13	-10.70	3.73	0.28
	25 mg, 10 µL	5 mg, 20 µL	-2.71	12.50	27.71	0.18
	25 mg, 10 µL	15 mg, 20 µL	-11.96	3.25	18.46	0.99
	25 mg, 10 µL	25 mg, 20 µL	-18.96	-3.75	11.46	0.98
	5 mg, 20 µL	15 mg, 20 µL	-24.46	-9.25	5.96	0.51
	5 mg, 20 µL	25 mg, 20 µL	-31.46	-16.25	-1.04	0.03
	15 mg, 20 µL	25 mg, 20 µL	-22.21	-7.00	8.21	0.78

Supplementary Table 3. Relative standard deviation (RSD in %) for individual compounds per experimental condition as measured by either liquid chromatography (LC) or flow injection analysis (FIA) mass spectrometry using Biocrates p400 HR kit in HOS cells. Five replicates were measured in each condition; outliers were removed from the final analysis as described in the materials and methods. NA: not applicable.

**Compound**	**Cell mass [mg]**	**5**	**15**	**25**	**5**	**15**	**25**
	**Loading volume [µL]**	**10**	**10**	**10**	**20**	**20**	**20**
AC(2:0)	Acylcarnitines	29.6	17.85	19.95	29.22	36.45	11.19
AC(3:1)	Acylcarnitines	NA	NA	NA	NA	NA	36.11
AC(4:0)	Acylcarnitines	NA	NA	NA	NA	10.46	NA
AC(5:0)	Acylcarnitines	NA	15.89	NA	21.61	22.21	NA
AC(5:0-DC)	Acylcarnitines	24.52	19.75	9.01	10.14	NA	NA
Ala	Amino acids	21.16	12.07	19	8.57	3.06	36.05
Arg	Amino acids	19.46	11.17	19.33	8.76	13.54	44.14
Asn	Amino acids	22.82	16.75	22.89	3.08	8.27	37.84
Asp	Amino acids	30.37	11.2	NA	14.25	7.24	NA
Gln	Amino acids	22.76	9.05	31.5	7.87	18.77	38.68
Glu	Amino acids	22.01	14.79	21.57	7.51	6.02	31.08
Gly	Amino acids	18.73	10.12	28.65	12.09	11.07	24.52
His	Amino acids	23.97	11.7	22.88	10.89	12.98	37.12
Ile	Amino acids	21.58	8.34	13.18	9.32	8.3	31.52
Lys	Amino acids	NA	NA	NA	NA	9.7	NA
Met	Amino acids	NA	19.76	NA	NA	7.48	NA
Orn	Amino acids	28.69	8.2	30.81	15.8	14.7	37.63
Phe	Amino acids	21.14	13.3	25.15	12.93	16.06	13.04
Pro	Amino acids	23.85	7.1	16.96	8.29	9.19	33.41
Ser	Amino acids	25.61	20.31	53.86	7.63	13.53	13.22

Thr	Amino acids	19.63	19.99	18.35	7.76	7.08	12.18
Trp	Amino acids	NA	18.08	NA	36.27	27.74	NA
Tyr	Amino acids	9.61	10.41	16.93	3.88	10.92	24.47
Val	Amino acids	10.05	4.91	14.96	12.87	13.93	18.02
xLeu	Amino acids	20.51	11.97	14.17	8.87	2.28	30.53
Creatinine	Biogenic amines	NA	2.43	NA	7.29	NA	NA
Met-SO	Biogenic amines	NA	NA	NA	NA	12.37	NA
Putrescine	Biogenic amines	24.36	20.79	18.99	10.22	7.81	21.06
Spermidine	Biogenic amines	14.38	24.48	14	9.15	6.15	23.7
Spermine	Biogenic amines	43.01	18.51	NA	46.66	13.23	32.53
t4-OH-Pro	Biogenic amines	22.55	15.49	24.25	1.73	1.74	32.53
Taurine	Biogenic amines	16.02	19.41	33.21	16.66	4.45	25.75
Thr	Biogenic amines	19.63	19.99	18.35	7.76	7.08	12.18
Cer(41:1)	Ceramides	NA	NA	NA	NA	NA	38.13
Cer(42:1)	Ceramides	NA	NA	11.11	NA	NA	NA
CE(18:1)	Cholesteryl esters	NA	NA	NA	NA	NA	18.2
CE(22:5)	Cholesteryl esters	14.31	13.78	38.65	28.03	19.17	32.38
CE(22:6)	Cholesteryl esters	19.53	12.92	33.05	38.17	32.51	49.42
DG(32:1)	Diglycerides	27.64	40.65	NA	24.05	18.29	39.27
DG(34:1)	Diglycerides	31.39	32	27.34	29.74	16.07	51.64
DG(36:2)	Diglycerides	20.93	27.85	11.76	17.64	16.47	28.81
DG(38:5)	Diglycerides	NA	NA	NA	35.03	22.17	NA
DG-O(34:1)	Diglycerides	21.2	16.04	13.17	23.57	13.41	15.22
LPC(14:0)	Lysophosphatidylcholines	NA	NA	0.69	NA	NA	NA
LPC(16:0)	Lysophosphatidylcholines	75.89	13.7	27.48	28.89	17.23	15.75
LPC(16:1)	Lysophosphatidylcholines	79.8	23.99	43.79	47.63	30.43	15.27
LPC(18:1)	Lysophosphatidylcholines	46.65	12.99	9.7	25.47	21.55	13.7
LPC(20:4)	Lysophosphatidylcholines	54.54	23.64	18.43	18.79	14.41	30.74
LPC(22:6)	Lysophosphatidylcholines	NA	NA	NA	46.27	30.9	NA
LPC(24:0)	Lysophosphatidylcholines	NA	NA	60.41	NA	NA	NA
LPC-O(18:0)	Lysophosphatidylcholines	NA	NA	NA	NA	25.43	NA
H1	Monosaccharides	NA	NA	NA	NA	3.75	NA
PC(25:0)	Phosphatidylcholines	81.33	21.34	14.22	NA	32.41	22.4
PC(26:0)	Phosphatidylcholines	NA	NA	4.72	NA	NA	NA
PC(27:1)	Phosphatidylcholines	42.92	NA	NA	NA	NA	NA
PC(29:0)	Phosphatidylcholines	24.24	11.25	NA	35.03	9.35	NA
PC(30:0)	Phosphatidylcholines	19.35	7.53	20.8	30.89	7.9	11.74
PC(30:1)	Phosphatidylcholines	17.72	12.39	21.53	33.5	4.25	12.66
PC(31:0)	Phosphatidylcholines	19.65	9.78	24.08	35.54	13.44	17.04
PC(31:1)	Phosphatidylcholines	19.14	8.5	19.47	37.74	10.45	14.76
PC(31:3)	Phosphatidylcholines	20.34	12.51	NA	15.87	12.15	NA
PC(32:0)	Phosphatidylcholines	11.73	18.29	27.1	29.61	13.45	29.33

PC(32:1)	Phosphatidylcholines	11.73	18.75	22.46	20.84	15.21	31.36
PC(32:2)	Phosphatidylcholines	19.51	26.44	15.42	25.84	15.89	34.62
PC(32:3)	Phosphatidylcholines	12.01	9.53	17.05	29.01	5.29	16.16
PC(32:4)	Phosphatidylcholines	14.78	7.98	17.72	31.09	7.29	20.64
PC(33:0)	Phosphatidylcholines	6.66	11.45	22.67	24.21	3.56	NA
PC(33:1)	Phosphatidylcholines	15.62	20.36	23.02	27.73	29.39	48.28
PC(33:2)	Phosphatidylcholines	15.3	11.98	29.97	35.82	8.59	27.01
PC(33:3)	Phosphatidylcholines	41.28	13.06	NA	25.4	3.82	24.54
PC(33:4)	Phosphatidylcholines	10.19	11.92	17.5	26.44	7.1	26.15
PC(34:0)	Phosphatidylcholines	94.74	28.49	19.17	62.25	NA	43.05
PC(34:1)	Phosphatidylcholines	10.97	8.66	25.05	28.99	8.63	25.51
PC(34:2)	Phosphatidylcholines	11.2	10.52	25.06	27.95	10.6	24.3
PC(34:3)	Phosphatidylcholines	7.04	17.64	24.45	36.43	9.2	27.22
PC(34:4)	Phosphatidylcholines	8.73	15.56	24.03	38.62	6.07	28.65
PC(34:5)	Phosphatidylcholines	8.37	17.7	25.09	38.45	5.74	33.67
PC(35:0)	Phosphatidylcholines	23.02	NA	NA	NA	NA	NA
PC(35:1)	Phosphatidylcholines	8.89	19.01	19.48	31.73	9.98	16.49
PC(35:2)	Phosphatidylcholines	9.91	12.02	23.27	34.55	6.98	34.79
PC(35:3)	Phosphatidylcholines	12.07	11.75	30.94	36.32	7.87	19.35
PC(35:4)	Phosphatidylcholines	10.85	19.12	23.82	37.13	9.31	23.2
PC(35:5)	Phosphatidylcholines	9.52	19.33	27.38	24.6	4.73	23.34
PC(36:0)	Phosphatidylcholines	NA	NA	NA	NA	NA	13.48
PC(36:1)	Phosphatidylcholines	10.5	28.33	26.43	32.64	11.52	30.98
PC(36:2)	Phosphatidylcholines	10.55	8.47	25.43	30.49	7.67	20.95
PC(36:3)	Phosphatidylcholines	8.57	13.77	20.44	35.69	6.7	23.89
PC(36:4)	Phosphatidylcholines	9.34	20.42	23.55	36.4	7.17	27.51
PC(36:5)	Phosphatidylcholines	9.66	15.42	24.35	37.48	5.44	26.73
PC(36:6)	Phosphatidylcholines	12.5	29.01	20.95	44.71	9.49	28.37
PC(37:1)	Phosphatidylcholines	10.32	19.53	14.02	30.44	16.85	17.65
PC(37:2)	Phosphatidylcholines	8.18	5.41	15.09	29.23	5.11	17.24
PC(37:3)	Phosphatidylcholines	8.76	18.28	20.48	35.09	8.99	20.36
PC(37:4)	Phosphatidylcholines	12.2	15.22	23.03	35.16	9.8	27.96
PC(37:5)	Phosphatidylcholines	21.63	22.68	26.75	35.64	5.59	33.11
PC(37:6)	Phosphatidylcholines	24.47	NA	NA	21.57	8.08	NA
PC(37:7)	Phosphatidylcholines	NA	NA	NA	3.89	8.36	NA
PC(38:0)	Phosphatidylcholines	36.66	15.93	11.82	29.76	28.72	NA
PC(38:1)	Phosphatidylcholines	14.83	22.37	22.66	21.68	7.16	NA
PC(38:2)	Phosphatidylcholines	14.32	9.37	20.95	33.79	9.2	19.21
PC(38:3)	Phosphatidylcholines	10.57	12.15	25.55	34.46	8.17	18.38
PC(38:4)	Phosphatidylcholines	10.62	17.9	26.66	36.03	7.87	28.04
PC(38:5)	Phosphatidylcholines	10.41	18.14	22.17	35.72	6.27	28.98
PC(38:6)	Phosphatidylcholines	10.45	14.18	22.77	37	5.86	25.82

PC(38:7)	Phosphatidylcholines	10.65	16.83	NA	22.8	4.98	NA
PC(39:0)	Phosphatidylcholines	7.8	20.72	6.6	19.47	6.63	8.01
PC(39:2)	Phosphatidylcholines	7.83	18.3	9.43	16.32	9.67	11.72
PC(39:3)	Phosphatidylcholines	16.43	18.85	12.28	14.04	5.94	14.92
PC(39:4)	Phosphatidylcholines	10.44	15.02	12.19	22.81	9.17	17.35
PC(39:5)	Phosphatidylcholines	13.43	25.49	25.94	37.63	9.19	21.71
PC(39:6)	Phosphatidylcholines	20.23	19.01	NA	27.17	7.42	NA
PC(39:7)	Phosphatidylcholines	9.52	NA	NA	22.83	11.51	NA
PC(40:1)	Phosphatidylcholines	12.09	20.47	14.23	25.82	38.86	8.15
PC(40:2)	Phosphatidylcholines	13.81	18.66	26.7	33.88	13.3	20.38
PC(40:3)	Phosphatidylcholines	12.67	9.42	19.5	35.88	10.06	14.22
PC(40:4)	Phosphatidylcholines	9.31	7.5	18.67	31.2	7.85	16.87
PC(40:5)	Phosphatidylcholines	11.38	13.46	24.1	34.97	7.92	24.4
PC(40:6)	Phosphatidylcholines	9.59	15.74	26.89	36.77	5.36	26.32
PC(40:7)	Phosphatidylcholines	11.57	14.72	26.62	36.38	6.1	25.54
PC(40:8)	Phosphatidylcholines	13.01	17.49	23.16	34.88	5.49	31.62
PC(40:9)	Phosphatidylcholines	12.94	14.15	23.12	38.45	7.01	33.61
PC(41:3)	Phosphatidylcholines	NA	NA	14.44	NA	NA	NA
PC(42:0)	Phosphatidylcholines	5.9	NA	25.35	13.15	5.95	NA
PC(42:1)	Phosphatidylcholines	NA	NA	NA	18.65	NA	NA
PC(42:10)	Phosphatidylcholines	NA	23.15	NA	43.94	2.1	20.9
PC(42:3)	Phosphatidylcholines	NA	NA	18.21	NA	NA	NA
PC(42:4)	Phosphatidylcholines	9.11	13.84	8.4	27.06	20.25	19.36
PC(42:5)	Phosphatidylcholines	9.82	24.89	45.35	26.92	19.31	10.78
PC(42:6)	Phosphatidylcholines	35.14	16.68	21.37	32.51	3.33	33.3
PC(42:7)	Phosphatidylcholines	14.2	10.77	14.64	33.93	8.18	19.14
PC(44:10)	Phosphatidylcholines	NA	22.99	NA	25.58	3.47	25.26
PC(44:3)	Phosphatidylcholines	20.55	NA	6.71	6.82	13.69	NA
PC(44:5)	Phosphatidylcholines	4.07	NA	18.55	29.91	18.31	NA
PC(44:6)	Phosphatidylcholines	21.69	NA	NA	23.24	15.49	NA
PC(44:7)	Phosphatidylcholines	11.26	NA	NA	23.02	17.17	NA
PC-O(30:0)	Phosphatidylcholines	14.88	7.89	21.3	31.71	8.41	16.11
PC-O(30:1)	Phosphatidylcholines	18.54	10.69	25.66	34.24	11.77	14.31
PC-O(30:2)	Phosphatidylcholines	NA	NA	NA	13.33	16.31	NA
PC-O(32:0)	Phosphatidylcholines	13.74	18.64	22.28	31.15	14.11	14.06
PC-O(32:1)	Phosphatidylcholines	14.92	6.22	23.12	33.96	7.25	16.7
PC-O(32:2)	Phosphatidylcholines	18.57	12.63	30.82	39.11	12.87	14.91
PC-O(32:3)	Phosphatidylcholines	18.59	8.29	25.67	29.33	6.94	17.63
PC-O(33:2)	Phosphatidylcholines	8.44	NA	NA	12.23	10.88	NA
PC-O(33:6)	Phosphatidylcholines	NA	NA	76.66	NA	NA	NA
PC-O(34:0)	Phosphatidylcholines	18.97	34.7	25.54	29.07	19.29	42.11
PC-O(34:1)	Phosphatidylcholines	10.31	7.51	26.25	32.89	8.62	31.36

PC-O(34:2)	Phosphatidylcholines	13.15	6.17	27.95	35.48	10.28	40.35
PC-O(34:3)	Phosphatidylcholines	9.67	26.21	26.57	38.49	12.71	40.69
PC-O(34:4)	Phosphatidylcholines	12.81	17.96	24.5	40.58	6.54	39.98
PC-O(35:3)	Phosphatidylcholines	11.84	NA	17.68	26.33	43.28	NA
PC-O(36:0)	Phosphatidylcholines	NA	NA	NA	19.93	NA	NA
PC-O(36:1)	Phosphatidylcholines	10.31	13.15	25.74	35.59	12.26	28.36
PC-O(36:2)	Phosphatidylcholines	13.41	13.52	26.22	33.86	8.82	30.74
PC-O(36:3)	Phosphatidylcholines	12.83	11.91	25.47	33.92	9.18	40.12
PC-O(36:4)	Phosphatidylcholines	11.52	19.97	24.93	38.86	8.33	26.52
PC-O(36:5)	Phosphatidylcholines	17.24	16.25	28.69	40.88	7.05	33.92
PC-O(36:6)	Phosphatidylcholines	2.31	26.26	NA	29.93	10.76	NA
PC-O(38:1)	Phosphatidylcholines	13.37	NA	22.3	28.62	18.76	38.19
PC-O(38:2)	Phosphatidylcholines	12.6	23.95	23.01	37.65	13.16	27.65
PC-O(38:3)	Phosphatidylcholines	12.12	16.97	30.02	39.64	11.99	31.24
PC-O(38:4)	Phosphatidylcholines	12.67	14.37	26.15	38.81	7.58	33.84
PC-O(38:5)	Phosphatidylcholines	14.09	13.3	28.06	40.06	6.9	40.41
PC-O(38:6)	Phosphatidylcholines	12.81	13.53	27.32	38.18	7.85	28.45
PC-O(40:1)	Phosphatidylcholines	17.34	NA	17.92	13.32	16.96	NA
PC-O(40:2)	Phosphatidylcholines	14.55	NA	25.79	30.4	17.92	21.5
PC-O(40:3)	Phosphatidylcholines	14.43	26.87	26.9	34.03	14.56	12.65
PC-O(40:4)	Phosphatidylcholines	14.79	18.32	27.04	36.5	11.24	25.08
PC-O(40:5)	Phosphatidylcholines	15.38	13.55	27.08	40.09	10.72	31.23
PC-O(40:6)	Phosphatidylcholines	14.75	19.44	30.72	40.53	8.13	33.04
PC-O(40:7)	Phosphatidylcholines	12.98	15.22	27.03	39.19	9.08	36.38
PC-O(40:8)	Phosphatidylcholines	21.94	16.21	34.18	41.95	7.08	35.25
PC-O(42:2)	Phosphatidylcholines	17.27	NA	9.21	31.7	22.84	NA
PC-O(42:3)	Phosphatidylcholines	17.65	NA	NA	24.57	17.26	NA
PC-O(42:4)	Phosphatidylcholines	12.2	NA	10.5	30.67	26.92	14.88
PC-O(42:5)	Phosphatidylcholines	35.63	NA	NA	31.56	10.99	17.3
PC-O(42:6)	Phosphatidylcholines	NA	NA	NA	39.35	3.47	14.64
PC-O(44:3)	Phosphatidylcholines	NA	NA	NA	33.43	19.8	NA
PC-O(44:4)	Phosphatidylcholines	7.49	NA	9.74	39.31	9.17	15.34
PC-O(44:5)	Phosphatidylcholines	8.64	NA	NA	24.83	NA	NA
SM(32:1)	Sphingomyelins	20.79	11.54	24.27	35.23	6.82	23.82
SM(33:1)	Sphingomyelins	16.75	8.09	25.3	29.69	21.28	17.05
SM(34:1)	Sphingomyelins	18.75	9.4	20.91	29.76	8.9	13.75
SM(34:2)	Sphingomyelins	26.94	21.12	47.77	30.42	9.65	12.39
SM(35:1)	Sphingomyelins	21.01	NA	8.25	34.18	9.38	NA
SM(36:0)	Sphingomyelins	13.26	NA	18.12	NA	NA	NA
SM(36:1)	Sphingomyelins	46.84	105.58	NA	9.87	7.46	NA
SM(36:2)	Sphingomyelins	NA	NA	NA	20.7	11.29	NA
SM(38:1)	Sphingomyelins	NA	NA	NA	19.63	NA	NA

SM(40:1)	Sphingomyelins	3.37	NA	23.97	18.59	23.77	15.13
SM(40:2)	Sphingomyelins	22.82	41.75	25.27	26.82	29.93	20.2
SM(41:1)	Sphingomyelins	10.07	NA	16.85	29.67	NA	17.71
SM(41:2)	Sphingomyelins	20.39	NA	57.15	14.47	11.73	NA
SM(42:1)	Sphingomyelins	10.19	33.16	19.44	28.23	17.04	17.25
SM(42:2)	Sphingomyelins	18.11	14.21	20.52	27.15	21.44	10.39
SM(42:3)	Sphingomyelins	17.36	9.68	19.99	24.16	20.21	18.37
SM(43:2)	Sphingomyelins	NA	NA	NA	7.69	13.8	NA
SM(44:2)	Sphingomyelins	15.09	NA	19.07	14.98	27.5	NA
TG(44:1)	Triglycerides	NA	NA	4.89	19.52	NA	NA
TG(48:1)	Triglycerides	NA	NA	NA	30.15	NA	NA
TG(49:1)	Triglycerides	NA	NA	NA	34.42	NA	NA
TG(49:2)	Triglycerides	NA	NA	3.46	NA	NA	NA
TG(50:1)	Triglycerides	NA	NA	NA	42.66	NA	27.99
TG(50:2)	Triglycerides	NA	NA	NA	43.77	NA	22.84
TG(50:3)	Triglycerides	27.93	35.08	5.81	53.24	NA	27.66
TG(52:2)	Triglycerides	NA	NA	NA	35.2	NA	15.57
TG(52:5)	Triglycerides	NA	NA	19.92	NA	NA	NA
TG(54:2)	Triglycerides	NA	NA	NA	NA	NA	26.01
TG(55:8)	Triglycerides	NA	NA	18.75	NA	NA	NA
Median RSD		14.55	15.56	22.825	29.76	9.7	24.81

Supplementary Table 4. Number of compounds detected for each cell lysis buffer used as measured by either liquid chromatography (LC) or flow injection analysis (FIA) mass spectrometry in HOS cells. Five replicates were measured in each condition; outliers were removed from the final analysis as described in the materials and methods.

Group	EtOH-P	EtOH	MeOH
Amino acids	20	21	20
Biogenic amines	6	6	6
Monosacchraides	1	1	1
Acylcarnitines	2	5	3
Diclycerides	1	3	4
Triglycerides	0	0	0
Lysophosphatidylcholines	3	7	5
Phosphatidylcholines	16	50	35
Sphingomyelins	1	3	6
Ceramides	0	0	0
Cholesteryl Esters	0	0	0

Supplementary table 5: Tukey Kramer post hoc statistical results of number of compounds detected in HOS cells for liquid chromatography (LC) and flow injection analysis (FIA) parts of the Biocrates analysis comparing different loading amounts.

Comparisons deemed significantly different are highlighted in bold. A Kruskal Wallis test was performed prior to the Tukey Kramer test. *CI = confidence interval.

	Comparison 1	Comparison 2	Lower limit of CI*	Estimated difference between group means	Upper limit of CI*	Tukey Kramer p value
LC	**EtOH-P**	**EtOH**	-11.3	-5.75	-0.191	0.041
	EtOH-P	MeOH	-6.31	-0.75	4.81	0.946
	EtOH	MeOH	-0.86	5	10.86	0.112
FIA	**EtOH-P**	**EtOH**	-13.95	-8	-2.05	0.0047
	EtOH-P	MeOH	-9.95	-4	1.95	0.257
	EtOH	MeOH	-1.95	4	9.95	0.257

Supplementary Table 6. Relative standard deviation (RSD in %) for individual compounds per condition as measured by either liquid chromatography (LC) or flow injection analysis (FIA) mass spectrometry in HOS cells. Five replicates were measured in each condition; outliers were removed from the final analysis as described in the materials and methods. NA: not applicable.

Compound	Class	EtOH-P	EtOH	MeOH
AC(0:0)	Acylcarnitines	NA	12	NA
AC(2:0)	Acylcarnitines	36	23	25
AC(4:0)	Acylcarnitines	NA	48	32
AC(5:0-DC)	Acylcarnitines	NA	55	NA
AC(5:0)	Diglycerides	32	22	28
Ala	Amino acids	30	5	12
Arg	Amino acids	19	10	8
Asn	Amino acids	39	6	14
Asp	Amino acids	38	13	15
Cit	Amino acids	NA	36	NA
Gln	Amino acids	40	7	13
Glu	Amino acids	41	8	23
Gly	Amino acids	21	10	7
His	Amino acids	21	14	8
Ile	Amino acids	24	11	9
Lys	Amino acids	11	15	14
Met	Amino acids	17	12	7
Orn	Amino acids	18	25	11
Phe	Amino acids	25	9	7
Pro	Amino acids	33	7	13
Ser	Amino acids	18	17	6
Thr	Amino acids	27	6	12
Trp	Amino acids	15	9	11

Tyr	Amino acids	20	9	5
Val	Amino acids	17	11	4
xLeu	Amino acids	17	7	5
Met-SO	Biogenic amines	8	22	9
Putrescine	Biogenic amines	57	8	34
Spermidine	Biogenic amines	36	6	10
Spermine	Biogenic amines	27	12	16
t4-OH-Pro	Biogenic amines	30	5	16
Taurine	Biogenic amines	39	3	29
DG-O(34:1)	Diglycerides	NA	28	24
DG(32:1)	Diglycerides	NA	19	31
DG(36:2)	Diglycerides	NA	NA	48
DG(42:2)	Diglycerides	23	36	33
H1	Hexose	17	14	10
LPC-O(16:1)	Lysophosphatidylcholines	NA	23	NA
LPC-O(18:0)	Lysophosphatidylcholines	NA	13	NA
LPC-O(18:1)	Lysophosphatidylcholines	NA	20	NA
LPC(16:0)	Lysophosphatidylcholines	NA	20	NA
LPC(16:1)	Lysophosphatidylcholines	12	31	17
LPC(18:1)	Lysophosphatidylcholines	18	20	13
LPC(18:2)	Lysophosphatidylcholines	NA	44	19
LPC(20:3)	Lysophosphatidylcholines	NA	23	6
LPC(20:4)	Lysophosphatidylcholines	16	19	16
LPC(22:6)	Lysophosphatidylcholines	NA	NA	17
PC-O(32:0)	Phosphatidylcholines	NA	13	46
PC-O(32:1)	Phosphatidylcholines	NA	7	NA
PC-O(32:2)	Phosphatidylcholines	NA	26	22
PC-O(32:3)	Phosphatidylcholines	NA	4	NA
PC-O(34:0)	Phosphatidylcholines	NA	NA	44
PC-O(34:1)	Phosphatidylcholines	NA	14	50
PC-O(34:2)	Phosphatidylcholines	NA	35	39
PC-O(34:3)	Phosphatidylcholines	NA	23	28
PC-O(34:4)	Phosphatidylcholines	NA	39	15
PC-O(36:1)	Phosphatidylcholines	NA	NA	35
PC-O(36:2)	Phosphatidylcholines	NA	17	48
PC-O(36:3)	Phosphatidylcholines	NA	54	NA
PC-O(36:4)	Phosphatidylcholines	NA	13	23
PC-O(36:5)	Phosphatidylcholines	NA	35	NA
PC-O(38:4)	Phosphatidylcholines	12	29	44
PC-O(38:5)	Phosphatidylcholines	NA	35	32
PC-O(38:6)	Phosphatidylcholines	NA	26	NA
PC-O(40:6)	Phosphatidylcholines	NA	53	NA
PC-O(40:7)	Phosphatidylcholines	NA	40	NA
PC-O(40:8)	Phosphatidylcholines	NA	21	NA
PC(30:0)	Phosphatidylcholines	NA	17	41
PC(30:1)	Phosphatidylcholines	NA	26	NA
PC(31:1)	Phosphatidylcholines	NA	17	NA

PC(32:0)	Phosphatidylcholines	66	32	53
PC(32:1)	Phosphatidylcholines	45	NA	47
PC(32:3)	Phosphatidylcholines	51	26	51
PC(32:4)	Phosphatidylcholines	NA	34	NA
PC(33:1)	Phosphatidylcholines	62	20	56
PC(33:2)	Phosphatidylcholines	NA	53	NA
PC(33:4)	Phosphatidylcholines	NA	26	NA
PC(34:1)	Phosphatidylcholines	80	12	53
PC(34:2)	Phosphatidylcholines	68	14	83
PC(34:3)	Phosphatidylcholines	NA	60	12
PC(34:4)	Phosphatidylcholines	42	13	25
PC(34:5)	Phosphatidylcholines	58	20	33
PC(35:2)	Phosphatidylcholines	NA	38	NA
PC(35:4)	Phosphatidylcholines	NA	51	NA
PC(35:5)	Phosphatidylcholines	NA	36	NA
PC(36:1)	Phosphatidylcholines	NA	48	20
PC(36:2)	Phosphatidylcholines	109	14	64
PC(36:4)	Phosphatidylcholines	49	34	30
PC(36:5)	Phosphatidylcholines	NA	16	15
PC(36:6)	Phosphatidylcholines	NA	39	NA
PC(37:5)	Phosphatidylcholines	NA	21	NA
PC(38:1)	Phosphatidylcholines	12		40
PC(38:2)	Phosphatidylcholines	NA	19	50
PC(38:3)	Phosphatidylcholines	NA	20	31
PC(38:4)	Phosphatidylcholines	36	35	27
PC(38:5)	Phosphatidylcholines	54	5	17
PC(38:6)	Phosphatidylcholines	48	20	37
PC(38:7)	Phosphatidylcholines	NA	21	NA
PC(39:3)	Phosphatidylcholines	NA	39	30
PC(39:4)	Phosphatidylcholines	NA	38	NA
PC(40:4)	Phosphatidylcholines	NA	36	NA
PC(40:5)	Phosphatidylcholines	NA	16	27
PC(40:6)	Phosphatidylcholines	NA	38	18
PC(40:7)	Phosphatidylcholines	NA	21	NA
PC(40:8)	Phosphatidylcholines	NA	27	NA
PC(42:10)	Phosphatidylcholines	NA	59	NA
PC(42:7)	Phosphatidylcholines	36	7	NA
SM(32:1)	Sphingomyelins	NA	6	NA
SM(34:1)	Sphingomyelins	62	14	45
SM(34:2)	Sphingomyelins	NA	21	NA
SM(38:3)	Sphingomyelins	NA	NA	19
SM(40:1)	Sphingomyelins	NA	NA	20
SM(42:1)	Sphingomyelins	NA	NA	26
SM(42:2)	Sphingomyelins	NA	NA	38
SM(42:3)	Sphingomyelins	NA	NA	25

Supplementary Table 7. The number of compounds detected, the median relative standard deviation (RSD) of the individual compounds and the percentage of compounds with a RSD < 15% threshold (in total and as a percentage of the total detected) for liquid chromatography (LC) and flow injection analysis (FIA) mass spectrometry analysis of MNNG/HOS and 143B cells. Five replicates were measured in each condition; outliers were removed from the final analysis as described in the materials and methods.

Cell line	Measure type	Lysis buffer	EtOH-P	EtOH	MeOH
HOS	LC	Number of compounds	26	27	26
		Median RSD [%]	25	9	11
		Number of compounds with RSD < 15%	12%	85%	88%
	FIA	Number of compounds	24	77	55
		Median RSD [%]	43	23	30
		Number of compounds with RSD < 15%	13%	21%	7%
MNNG/HOS	LC	Number of compounds	25	27	21
		Median RSD [%]	18	8	21
		Number of compounds with RSD < 15%	28	74	17
	FIA	Number of compounds	50	114	33
		Median RSD [%]	36	15	32
		Number of compounds with RSD < 15%	12	53	3
143B	LC	Number of compounds	28	28	25
		Median RSD [%]	24	21	14
		Number of compounds with RSD < 15%	7	14	56
	FIA	Number of compounds	14	128	39
		Median RSD [%]	62	23	71
		Number of compounds with RSD < 15%	0	5	3

Supplementary Table 8. Compounds measured with mass spectrometry using the Absolute*IDQ* p400 HR kit. LC: Liquid chromatography. FIA: Flow injection analysis.

Compound	Name	Class	Method
Ala	Alanine	Amino Acids	LC
Lys	Lysine	Amino Acids	LC
Arg	Arginine	Amino Acids	LC
Met	Methionine	Amino Acids	LC
Asn	Asparagine	Amino Acids	LC
Orn	Ornithine	Amino Acids	LC
Asp	Aspartate	Amino Acids	LC
Phe	Phenylalanine	Amino Acids	LC

Cit	Citrulline	Amino Acids	LC
Pro	Proline	Amino Acids	LC
Glu	Glutamate	Amino Acids	LC
Ser	Serine	Amino Acids	LC
Gln	Glutamine	Amino Acids	LC
Thr	Threonine	Amino Acids	LC
Gly	Glycine	Amino Acids	LC
Trp	Tryptophan	Amino Acids	LC
His	Histidine	Amino Acids	LC
Tyr	Tyrosine	Amino Acids	LC
Ile	Isoleucine	Amino Acids	LC
Val	Valine	Amino Acids	LC
xLeu	Leucine + Isoleucine	Amino Acids	LC
Ac-Orn	Acetylornithine	Biogenic Amines	LC
Nitro-Tyr	Nitrotyrosine	Biogenic Amines	LC
ADMA	Asymmetric dimethylarginine	Biogenic Amines	LC
PEA	Phenylethylamine	Biogenic Amines	LC
alpha-AAA	alpha-Aminoadipic acid	Biogenic Amines	LC
Putrescine	Putrescine	Biogenic Amines	LC
Carnosine	Carnosine	Biogenic Amines	LC
Sarcosine	Sarcosine	Biogenic Amines	LC
c4-OH-Pro	cis-4-Hydroxyproline	Biogenic Amines	LC
SDMA	Symmetric dimethylarginine	Biogenic Amines	LC
Creatinine	Creatinine	Biogenic Amines	LC
Serotonin	Serotonin	Biogenic Amines	LC
DOPA	Dihydroxyphenylalanine	Biogenic Amines	LC
Spermidine	Spermidine	Biogenic Amines	LC
Dopamine	Dopamine	Biogenic Amines	LC
Spermine	Spermine	Biogenic Amines	LC
Histamine	Histamine	Biogenic Amines	LC
Taurine	Taurine	Biogenic Amines	LC
Kynurenine	Kynurenine	Biogenic Amines	LC
t4-OH-Pro	trans-Hydroxyproline	Biogenic Amines	LC
Met-SO	Methionine sulfoxide	Biogenic Amines	LC
H1	Hexoses (including glucose)	Monosaccharides	FIA
AC(0:0)	Carnitine	Acylcarnitines	FIA
AC(4:1-DC)	Fumarylcarnitine	Acylcarnitines	FIA
AC(2:0)	Acetylcarnitine	Acylcarnitines	FIA
AC(5:0)	Valerylcarnitine	Acylcarnitines	FIA
AC(3:0)	Propionylcarnitine	Acylcarnitines	FIA
AC(5:0-DC)	Glutarylcarnitine	Acylcarnitines	FIA
AC(3:0-DC)	Malonylcarnitine	Acylcarnitines	FIA

AC(5:0-OH)	Hydroxyvalerylcarnitine	Acylcarnitines	FIA
AC(3:0-OH)	Hydroxypropionylcarnitine	Acylcarnitines	FIA
AC(5:1)	Tiglylcarnitine	Acylcarnitines	FIA
AC(3:1)	Propenoylcarnitine	Acylcarnitines	FIA
AC(5:1-DC)	Glutaconylcarnitine	Acylcarnitines	FIA
AC(4:0)	Butyrylcarnitine	Acylcarnitines	FIA
AC(6:0)	Hexanoylcarnitine	Acylcarnitines	FIA
AC(4:0-DC)	Methylmalonylcarnitine	Acylcarnitines	FIA
AC(6:0-DC)	Adipoylcarnitine	Acylcarnitines	FIA
AC(4:0-OH)	Hydroxybutyrylcarnitine	Acylcarnitines	FIA
AC(6:0-OH)	Hydroxyhexanoylcarnitine	Acylcarnitines	FIA
AC(4:1)	Butenylcarnitine	Acylcarnitines	FIA
AC(6:1)	Hexenoylcarnitine	Acylcarnitines	FIA
AC(7:0)	Heptanoylcarnitine	Acylcarnitines	FIA
AC(14:1-DC)	Carboxytridecenoylcarnitine	Acylcarnitines	FIA
AC(7:0-DC)	Pimeloylcarnitine	Acylcarnitines	FIA
AC(14:1-OH)	Hydroxytetradecenoylcarnitine	Acylcarnitines	FIA
AC(8:0)	Octanoylcarnitine	Acylcarnitines	FIA
AC(14:2)	Tetradecadienoylcarnitine	Acylcarnitines	FIA
AC(8:1)	Octenoylcarnitine	Acylcarnitines	FIA
AC(14:2-OH)	Hydroxytetradecadienoylcarnitine	Acylcarnitines	FIA
AC(8:1-OH)	Hydroxyoctenoylcarnitine	Acylcarnitines	FIA
AC(15:0)	Pentadecanoylcarnitine	Acylcarnitines	FIA
AC(9:0)	Nonaylcarnitine	Acylcarnitines	FIA
AC(16:0)	Hexadecanoylcarnitine	Acylcarnitines	FIA
AC(10:0)	Decanoylcarnitine	Acylcarnitines	FIA
AC(16:0-OH)	Hydroxyhexadecanoylcarnitine	Acylcarnitines	FIA
AC(10:1)	Decenoylcarnitine	Acylcarnitines	FIA
AC(16:1)	Hexadecenoylcarnitine	Acylcarnitines	FIA
AC(10:2)	Decadienoylcarnitine	Acylcarnitines	FIA
AC(16:1-OH)	Hydroxyhexadecenoylcarnitine	Acylcarnitines	FIA
AC(10:3)	Decatrienoylcarnitine	Acylcarnitines	FIA
AC(16:2)	Hexadecadienoylcarnitine	Acylcarnitines	FIA
AC(11:0)	Dimethylnonanoylcarnitine	Acylcarnitines	FIA
AC(16:2-OH)	Hydroxyhexadecadienoylcarnitine	Acylcarnitines	FIA
AC(12:0)	Dodecanoylcarnitine	Acylcarnitines	FIA
AC(17:0)	Heptadecanoylcarnitine	Acylcarnitines	FIA
AC(12:0-DC)	Dodecanedioylcarnitine	Acylcarnitines	FIA
AC(18:0)	Octadecanoylcarnitine	Acylcarnitines	FIA
AC(12:1)	Dodecenoylcarnitine	Acylcarnitines	FIA
AC(18:1)	Octadecenoylcarnitine	Acylcarnitines	FIA
AC(13:0)	Tridecanoylcarnitine	Acylcarnitines	FIA

AC(18:1-OH)	Hydroxyoctadecenoylcarnitine	Acylcarnitines	FIA
AC(14:0)	Tetradecanoylcarnitine	Acylcarnitines	FIA
AC(18:2)	Octadecadienylcarnitine	Acylcarnitines	FIA
AC(14:0-OH)	Hydroxymyristoylcarnitine	Acylcarnitines	FIA
AC(19:0)	Nonadecanoylcarnitine	Acylcarnitines	FIA
AC(14:1)	Tetradecenoylcarnitine	Acylcarnitines	FIA
DG(32:1)		Diglycerides	FIA
DG(36:3)		Diglycerides	FIA
DG(41:1)		Diglycerides	FIA
DG-O(32:2)		Diglycerides	FIA
DG(32:2)		Diglycerides	FIA
DG(36:4)		Diglycerides	FIA
DG(42:0)		Diglycerides	FIA
DG-O(34:1)		Diglycerides	FIA
DG(34:1)		Diglycerides	FIA
DG(38:0)		Diglycerides	FIA
DG(42:1)		Diglycerides	FIA
DG-O(36:4)		Diglycerides	FIA
DG(34:3)		Diglycerides	FIA
DG(38:5)		Diglycerides	FIA
DG(42:2)		Diglycerides	FIA
DG(36:2)		Diglycerides	FIA
DG(39:0)		Diglycerides	FIA
DG(44:3)		Diglycerides	FIA
TG(44:1)		Triglycerides	FIA
TG(50:3)		Triglycerides	FIA
TG(52:6)		Triglycerides	FIA
TG(54:7)		Triglycerides	FIA
TG(44:2)		Triglycerides	FIA
TG(50:4)		Triglycerides	FIA
TG(52:7)		Triglycerides	FIA
TG(55:6)		Triglycerides	FIA
TG(44:4)		Triglycerides	FIA
TG(51:1)		Triglycerides	FIA
TG(53:3)		Triglycerides	FIA
TG(55:7)		Triglycerides	FIA
TG(46:2)		Triglycerides	FIA
TG(51:2)		Triglycerides	FIA
TG(53:4)		Triglycerides	FIA
TG(55:8)		Triglycerides	FIA
TG(48:1)		Triglycerides	FIA
TG(51:3)		Triglycerides	FIA

TG(53:5)		Triglycerides	FIA
TG(55:9)		Triglycerides	FIA
TG(48:2)		Triglycerides	FIA
TG(51:4)		Triglycerides	FIA
TG(53:6)		Triglycerides	FIA
TG(56:6)		Triglycerides	FIA
TG(48:3)		Triglycerides	FIA
TG(51:5)		Triglycerides	FIA
TG(54:2)		Triglycerides	FIA
TG(56:7)		Triglycerides	FIA
TG(49:1)		Triglycerides	FIA
TG(52:2)		Triglycerides	FIA
TG(54:3)		Triglycerides	FIA
TG(56:8)		Triglycerides	FIA
TG(49:2)		Triglycerides	FIA
TG(52:3)		Triglycerides	FIA
TG(54:4)		Triglycerides	FIA
TG(56:9)		Triglycerides	FIA
TG(50:1)		Triglycerides	FIA
TG(52:4)		Triglycerides	FIA
TG(54:5)		Triglycerides	FIA
TG(50:2)		Triglycerides	FIA
TG(52:5)		Triglycerides	FIA
TG(54:6)		Triglycerides	FIA
LPC(12:0)		Lysophosphatidylcholines	FIA
LPC(17:1)		Lysophosphatidylcholines	FIA
LPC(20:2)		Lysophosphatidylcholines	FIA
LPC(24:1)		Lysophosphatidylcholines	FIA
LPC(14:0)		Lysophosphatidylcholines	FIA
LPC(18:0)		Lysophosphatidylcholines	FIA
LPC(20:3)		Lysophosphatidylcholines	FIA
LPC-O(16:1)		Lysophosphatidylcholines	FIA
LPC(15:0)		Lysophosphatidylcholines	FIA
LPC(18:1)		Lysophosphatidylcholines	FIA
LPC(20:4)		Lysophosphatidylcholines	FIA
LPC-O(17:1)		Lysophosphatidylcholines	FIA
LPC(16:0)		Lysophosphatidylcholines	FIA
LPC(18:2)		Lysophosphatidylcholines	FIA
LPC(22:5)		Lysophosphatidylcholines	FIA
LPC-O(18:0)		Lysophosphatidylcholines	FIA
LPC(16:1)		Lysophosphatidylcholines	FIA
LPC(20:0)		Lysophosphatidylcholines	FIA

LPC(22:6)		Lysophosphatidylcholines	FIA
LPC-O(18:1)		Lysophosphatidylcholines	FIA
LPC(17:0)		Lysophosphatidylcholines	FIA
LPC(20:1)		Lysophosphatidylcholines	FIA
LPC(24:0)		Lysophosphatidylcholines	FIA
LPC-O(18:2)		Lysophosphatidylcholines	FIA
PC(24:0)		Lysophosphatidylcholines	FIA
PC(32:6)		Lysophosphatidylcholines	FIA
PC(36:4)		Phosphatidylcholines	FIA
PC(39:4)		Phosphatidylcholines	FIA
PC(25:0)		Phosphatidylcholines	FIA
PC(33:0)		Phosphatidylcholines	FIA
PC(36:5)		Phosphatidylcholines	FIA
PC(39:5)		Phosphatidylcholines	FIA
PC(26:0)		Phosphatidylcholines	FIA
PC(33:1)		Phosphatidylcholines	FIA
PC(36:6)		Phosphatidylcholines	FIA
PC(39:6)		Phosphatidylcholines	FIA
PC(27:0)		Phosphatidylcholines	FIA
PC(33:2)		Phosphatidylcholines	FIA
PC(37:0)		Phosphatidylcholines	FIA
PC(39:7)		Phosphatidylcholines	FIA
PC(27:1)		Phosphatidylcholines	FIA
PC(33:3)		Phosphatidylcholines	FIA
PC(37:1)		Phosphatidylcholines	FIA
PC(40:1)		Phosphatidylcholines	FIA
PC(28:1)		Phosphatidylcholines	FIA
PC(33:4)		Phosphatidylcholines	FIA
PC(37:2)		Phosphatidylcholines	FIA
PC(40:2)		Phosphatidylcholines	FIA
PC(29:0)		Phosphatidylcholines	FIA
PC(33:5)		Phosphatidylcholines	FIA
PC(37:3)		Phosphatidylcholines	FIA
PC(40:3)		Phosphatidylcholines	FIA
PC(29:1)		Phosphatidylcholines	FIA
PC(34:0)		Phosphatidylcholines	FIA
PC(37:4)		Phosphatidylcholines	FIA
PC(40:4)		Phosphatidylcholines	FIA
PC(29:2)		Phosphatidylcholines	FIA
PC(34:1)		Phosphatidylcholines	FIA
PC(37:5)		Phosphatidylcholines	FIA
PC(40:5)		Phosphatidylcholines	FIA

PC(30:0)		Phosphatidylcholines	FIA
PC(34:2)		Phosphatidylcholines	FIA
PC(37:6)		Phosphatidylcholines	FIA
PC(40:6)		Phosphatidylcholines	FIA
PC(30:1)		Phosphatidylcholines	FIA
PC(34:3)		Phosphatidylcholines	FIA
PC(37:7)		Phosphatidylcholines	FIA
PC(40:7)		Phosphatidylcholines	FIA
PC(30:2)		Phosphatidylcholines	FIA
PC(34:4)		Phosphatidylcholines	FIA
PC(38:0)		Phosphatidylcholines	FIA
PC(40:8)		Phosphatidylcholines	FIA
PC(30:3)		Phosphatidylcholines	FIA
PC(34:5)		Phosphatidylcholines	FIA
PC(38:1)		Phosphatidylcholines	FIA
PC(40:9)		Phosphatidylcholines	FIA
PC(31:0)		Phosphatidylcholines	FIA
PC(35:0)		Phosphatidylcholines	FIA
PC(38:2)		Phosphatidylcholines	FIA
PC(41:1)		Phosphatidylcholines	FIA
PC(31:1)		Phosphatidylcholines	FIA
PC(35:1)		Phosphatidylcholines	FIA
PC(38:3)		Phosphatidylcholines	FIA
PC(41:2)		Phosphatidylcholines	FIA
PC(31:2)		Phosphatidylcholines	FIA
PC(35:2)		Phosphatidylcholines	FIA
PC(38:4)		Phosphatidylcholines	FIA
PC(41:3)		Phosphatidylcholines	FIA
PC(31:3)		Phosphatidylcholines	FIA
PC(35:3)		Phosphatidylcholines	FIA
PC(38:5)		Phosphatidylcholines	FIA
PC(41:4)		Phosphatidylcholines	FIA
PC(32:0)		Phosphatidylcholines	FIA
PC(35:4)		Phosphatidylcholines	FIA
PC(38:6)		Phosphatidylcholines	FIA
PC(41:5)		Phosphatidylcholines	FIA
PC(32:1)		Phosphatidylcholines	FIA
PC(35:5)		Phosphatidylcholines	FIA
PC(38:7)		Phosphatidylcholines	FIA
PC(41:8)		Phosphatidylcholines	FIA
PC(32:2)		Phosphatidylcholines	FIA
PC(36:0)		Phosphatidylcholines	FIA

PC(39:0)		Phosphatidylcholines	FIA
PC(42:0)		Phosphatidylcholines	FIA
PC(32:3)		Phosphatidylcholines	FIA
PC(36:1)		Phosphatidylcholines	FIA
PC(39:1)		Phosphatidylcholines	FIA
PC(42:1)		Phosphatidylcholines	FIA
PC(32:4)		Phosphatidylcholines	FIA
PC(36:2)		Phosphatidylcholines	FIA
PC(39:2)		Phosphatidylcholines	FIA
PC(42:2)		Phosphatidylcholines	FIA
PC(32:5)		Phosphatidylcholines	FIA
PC(36:3)		Phosphatidylcholines	FIA
PC(39:3)		Phosphatidylcholines	FIA
PC(42:3)		Phosphatidylcholines	FIA
PC(42:4)		Phosphatidylcholines	FIA
PC-O(29:0)		Phosphatidylcholines	FIA
PC-O(34:3)		Phosphatidylcholines	FIA
PC-O(40:0)		Phosphatidylcholines	FIA
PC(42:5)		Phosphatidylcholines	FIA
PC-O(30:0)		Phosphatidylcholines	FIA
PC-O(34:4)		Phosphatidylcholines	FIA
PC-O(40:1)		Phosphatidylcholines	FIA
PC(42:6)		Phosphatidylcholines	FIA
PC-O(30:1)		Phosphatidylcholines	FIA
PC-O(35:3)		Phosphatidylcholines	FIA
PC-O(40:2)		Phosphatidylcholines	FIA
PC(42:7)		Phosphatidylcholines	FIA
PC-O(30:2)		Phosphatidylcholines	FIA
PC-O(35:4)		Phosphatidylcholines	FIA
PC-O(40:3)		Phosphatidylcholines	FIA
PC(42:10)		Phosphatidylcholines	FIA
PC-O(31:0)		Phosphatidylcholines	FIA
PC-O(36:0)		Phosphatidylcholines	FIA
PC-O(40:4)		Phosphatidylcholines	FIA
PC(43:2)		Phosphatidylcholines	FIA
PC-O(31:1)		Phosphatidylcholines	FIA
PC-O(36:1)		Phosphatidylcholines	FIA
PC-O(40:5)		Phosphatidylcholines	FIA
PC(43:6)		Phosphatidylcholines	FIA
PC-O(31:3)		Phosphatidylcholines	FIA
PC-O(36:2)		Phosphatidylcholines	FIA
PC-O(40:6)		Phosphatidylcholines	FIA

PC(44:1)		Phosphatidylcholines	FIA
PC-O(32:0)		Phosphatidylcholines	FIA
PC-O(36:3)		Phosphatidylcholines	FIA
PC-O(40:7)		Phosphatidylcholines	FIA
PC(44:3)		Phosphatidylcholines	FIA
PC-O(32:1)		Phosphatidylcholines	FIA
PC-O(36:4)		Phosphatidylcholines	FIA
PC-O(40:8)		Phosphatidylcholines	FIA
PC(44:5)		Phosphatidylcholines	FIA
PC-O(32:2)		Phosphatidylcholines	FIA
PC-O(36:5)		Phosphatidylcholines	FIA
PC-O(42:0)		Phosphatidylcholines	FIA
PC(44:6)		Phosphatidylcholines	FIA
PC-O(32:3)		Phosphatidylcholines	FIA
PC-O(36:6)		Phosphatidylcholines	FIA
PC-O(42:1)		Phosphatidylcholines	FIA
PC(44:7)		Phosphatidylcholines	FIA
PC-O(33:0)		Phosphatidylcholines	FIA
PC-O(37:6)		Phosphatidylcholines	FIA
PC-O(42:2)		Phosphatidylcholines	FIA
PC(44:10)		Phosphatidylcholines	FIA
PC-O(33:1)		Phosphatidylcholines	FIA
PC-O(37:7)		Phosphatidylcholines	FIA
PC-O(42:3)		Phosphatidylcholines	FIA
PC(44:12)		Phosphatidylcholines	FIA
PC-O(33:2)		Phosphatidylcholines	FIA
PC-O(38:0)		Phosphatidylcholines	FIA
PC-O(42:4)		Phosphatidylcholines	FIA
PC(46:1)		Phosphatidylcholines	FIA
PC-O(33:3)		Phosphatidylcholines	FIA
PC-O(38:1)		Phosphatidylcholines	FIA
PC-O(42:5)		Phosphatidylcholines	FIA
PC(46:2)		Phosphatidylcholines	FIA
PC-O(33:4)		Phosphatidylcholines	FIA
PC-O(38:2)		Phosphatidylcholines	FIA
PC-O(42:6)		Phosphatidylcholines	FIA
PC-O(26:0)		Phosphatidylcholines	FIA
PC-O(33:6)		Phosphatidylcholines	FIA
PC-O(38:3)		Phosphatidylcholines	FIA
PC-O(44:3)		Phosphatidylcholines	FIA
PC-O(26:1)		Phosphatidylcholines	FIA
PC-O(34:0)		Phosphatidylcholines	FIA

PC-O(38:4)		Phosphatidylcholines	FIA
PC-O(44:4)		Phosphatidylcholines	FIA
PC-O(28:0)		Phosphatidylcholines	FIA
PC-O(34:1)		Phosphatidylcholines	FIA
PC-O(38:5)		Phosphatidylcholines	FIA
PC-O(44:5)		Phosphatidylcholines	FIA
PC-O(28:1)		Phosphatidylcholines	FIA
PC-O(34:2)		Phosphatidylcholines	FIA
PC-O(38:6)		Phosphatidylcholines	FIA
PC-O(44:6)		Phosphatidylcholines	FIA
SM(30:1)		Sphingomyelins	FIA
SM(34:2)		Sphingomyelins	FIA
SM(38:3)		Sphingomyelins	FIA
SM(42:1)		Sphingomyelins	FIA
SM(31:0)		Sphingomyelins	FIA
SM(35:1)		Sphingomyelins	FIA
SM(39:1)		Sphingomyelins	FIA
SM(42:2)		Sphingomyelins	FIA
SM(31:1)		Sphingomyelins	FIA
SM(36:0)		Sphingomyelins	FIA
SM(39:2)		Sphingomyelins	FIA
SM(42:3)		Sphingomyelins	FIA
SM(32:1)		Sphingomyelins	FIA
SM(36:1)		Sphingomyelins	FIA
SM(40:1)		Sphingomyelins	FIA
SM(43:1)		Sphingomyelins	FIA
SM(32:2)		Sphingomyelins	FIA
SM(36:2)		Sphingomyelins	FIA
SM(40:2)		Sphingomyelins	FIA
SM(43:2)		Sphingomyelins	FIA
SM(33:1)		Sphingomyelins	FIA
SM(37:1)		Sphingomyelins	FIA
SM(40:4)		Sphingomyelins	FIA
SM(44:1)		Sphingomyelins	FIA
SM(33:2)		Sphingomyelins	FIA
SM(38:1)		Sphingomyelins	FIA
SM(41:1)		Sphingomyelins	FIA
SM(44:2)		Sphingomyelins	FIA
SM(34:1)		Sphingomyelins	FIA
SM(38:2)		Sphingomyelins	FIA
SM(41:2)		Sphingomyelins	FIA
Cer(34:0)		Ceramides	FIA

Cer(40:1)		Ceramides	FIA
Cer(42:2)		Ceramides	FIA
Cer(34:1)		Ceramides	FIA
Cer(41:1)		Ceramides	FIA
Cer(43:1)		Ceramides	FIA
Cer(38:1)		Ceramides	FIA
Cer(42:1)		Ceramides	FIA
Cer(44:0)		Ceramides	FIA
CE(16:0)		Cholesteryl Esters	FIA
CE(17:2)		Cholesteryl Esters	FIA
CE(19:2)		Cholesteryl Esters	FIA
CE(22:5)		Cholesteryl Esters	FIA
CE(16:1)		Cholesteryl Esters	FIA
CE(18:1)		Cholesteryl Esters	FIA
CE(19:3)		Cholesteryl Esters	FIA
CE(22:6)		Cholesteryl Esters	FIA
CE(17:0)		Cholesteryl Esters	FIA
CE(18:2)		Cholesteryl Esters	FIA
CE(20:4)		Cholesteryl Esters	FIA
CE(17:1)		Cholesteryl Esters	FIA
CE(18:3)		Cholesteryl Esters	FIA
CE(20:5)		Cholesteryl Esters	FIA

Supplementary Table 9. UHPLC gradient for liquid chromatography measurement 1 and 2.

Number	Time [min]	Flow [mL/min]	B [%]	Curve
1	0.00	Run		
2	0.00	0.8	0.0	5
3	0.25	0.8	0.0	5
4	1.50	0.8	12.0	5
5	2.70	0.8	17.5	5
6	4.00	0.8	50.0	5
7	4.50	0.8	95.0	5
8	4.70	1.0	95.0	5
9	5.10	1.0	95.0	5
10	5.25	1.0	0.0	5
11	6.80	0.8	0.0	5
12	6.81	Stop run		

Supplementary Table 10. FIA gradient for flow injection analysis measurement 1 and 2.

Number	Time [min]	Flow [mL/min]	B [%]	Curve
1	0.00	Run		
2	0.00	0.05	0.0	5
3	1.40	0.05	0.0	5
4	1.60	0.20	0.0	5
5	2.80	0.20	0.0	5
6	3.00	0.05	0.0	5
7	3.01	Stop run		

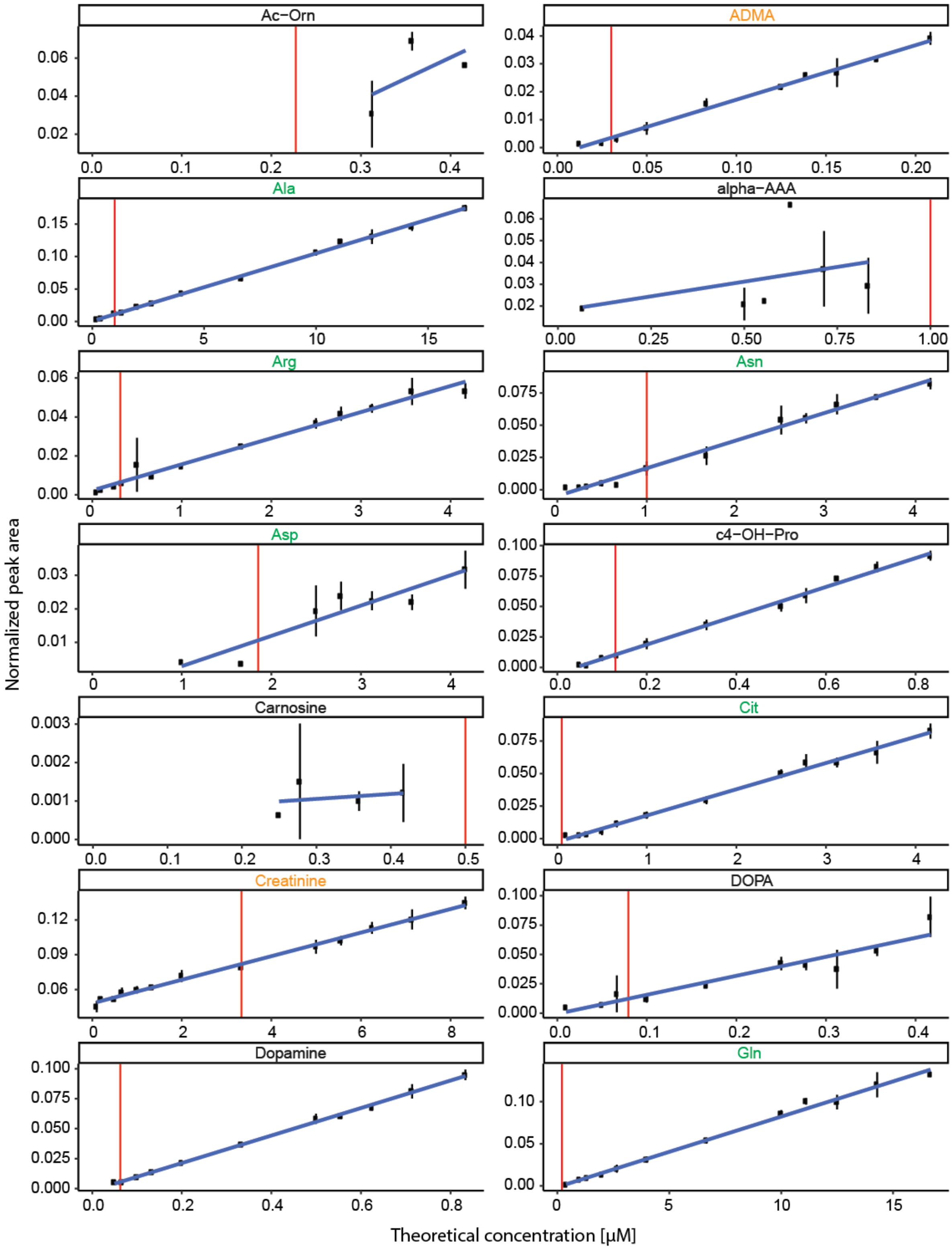
Ac−Orn
ADMA
Ala
alpha−AAA
Arg
Asn
Asp
c4−OH−Pro
Carnosine
Cit
Creatinine
DOPA
Dopamine
Gln
Normalized peak area
Theoretical concentration [µM]

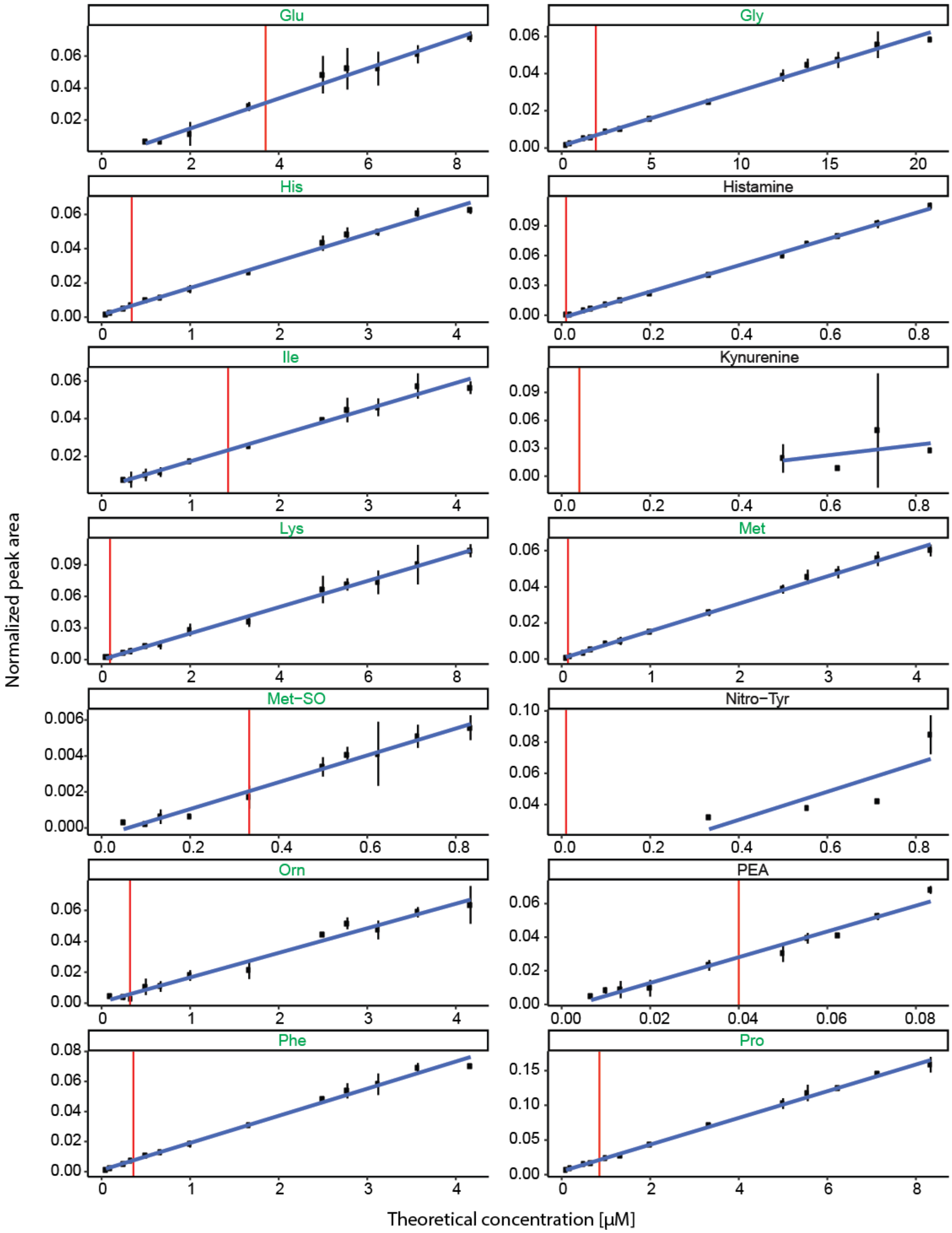

Glu
Gly
His
Histamine
Ile
Kynurenine
Lys
Met
Met−SO
Nitro−Tyr
Orn
PEA
Phe
Pro
Normalized peak area
Theoretical concentration [µM]

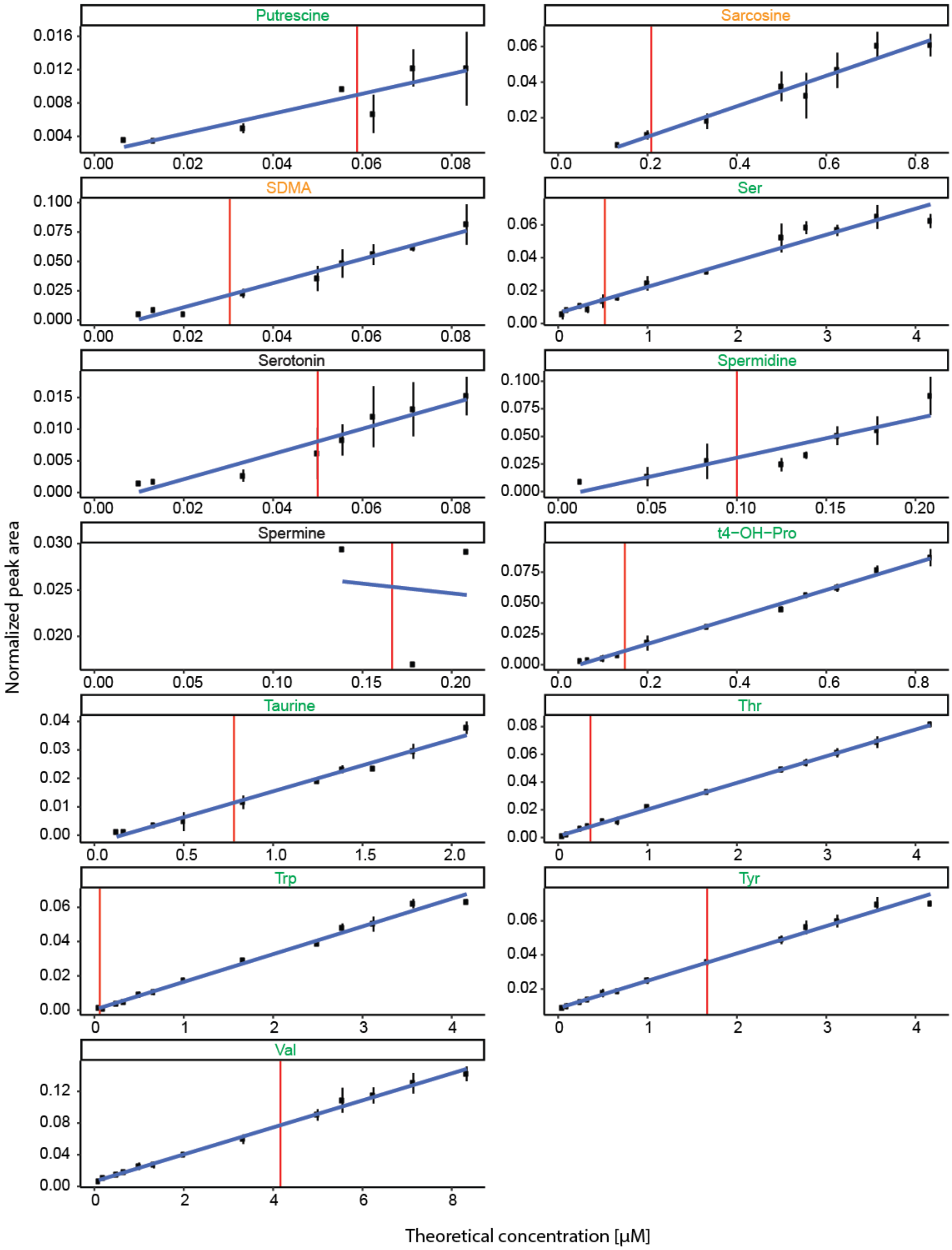

Supplementary Figure 1. Line graphs of normalized measured peak area against theoretical concentration of individual compounds found in the dilution series of the calibration 1 (Cal 1) solution provided by Biocrates. Each data point has been acquired in triplicate, the error bars represent the standard deviation of the normalized peak area (represented by black dots). The vertical red line represents the concentration at which Biocrates by default has established the limit of detection threshold

(LOD). A linear regression curve (blue line) is shown for visualization purposes only. No distinction is made between missing data points due to a lack of detection of a compound or of its associated internal standard. The colours of the metabolite names indicate the performance of that metabolite when measured in adherent HOS cells. Green represents compounds where the normalized measured peak area falls within the Bioacrates calibration range, in orange are the compounds below the Biocrates calibration range but within our diluted calibration range. Black compounds are detected but above the LLOQ.

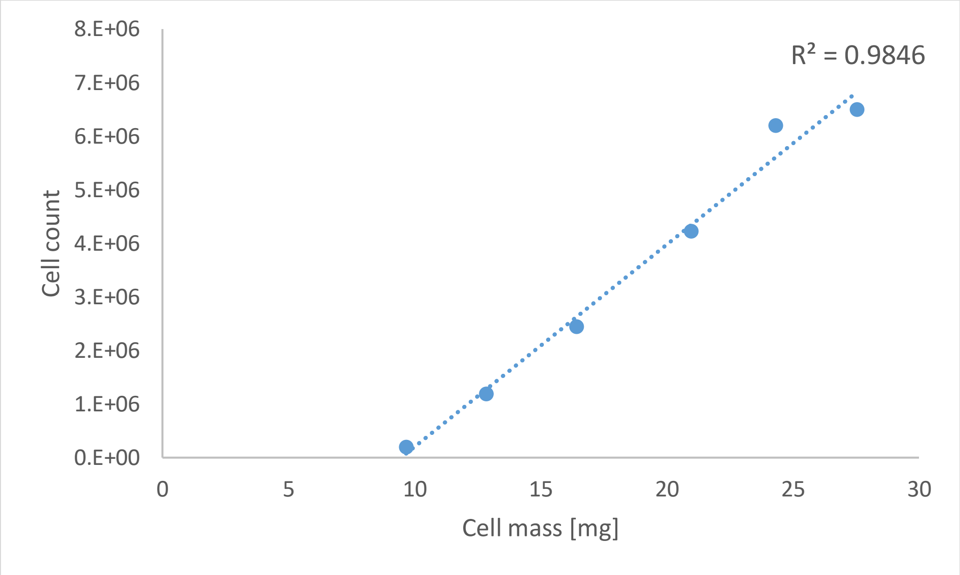

Supplementary Figure 2. Relationship between cell mass and cell count in HOS osteosarcoma cells. The mean of three measured biological replicates is shown. Linear regression curve is overlaid.

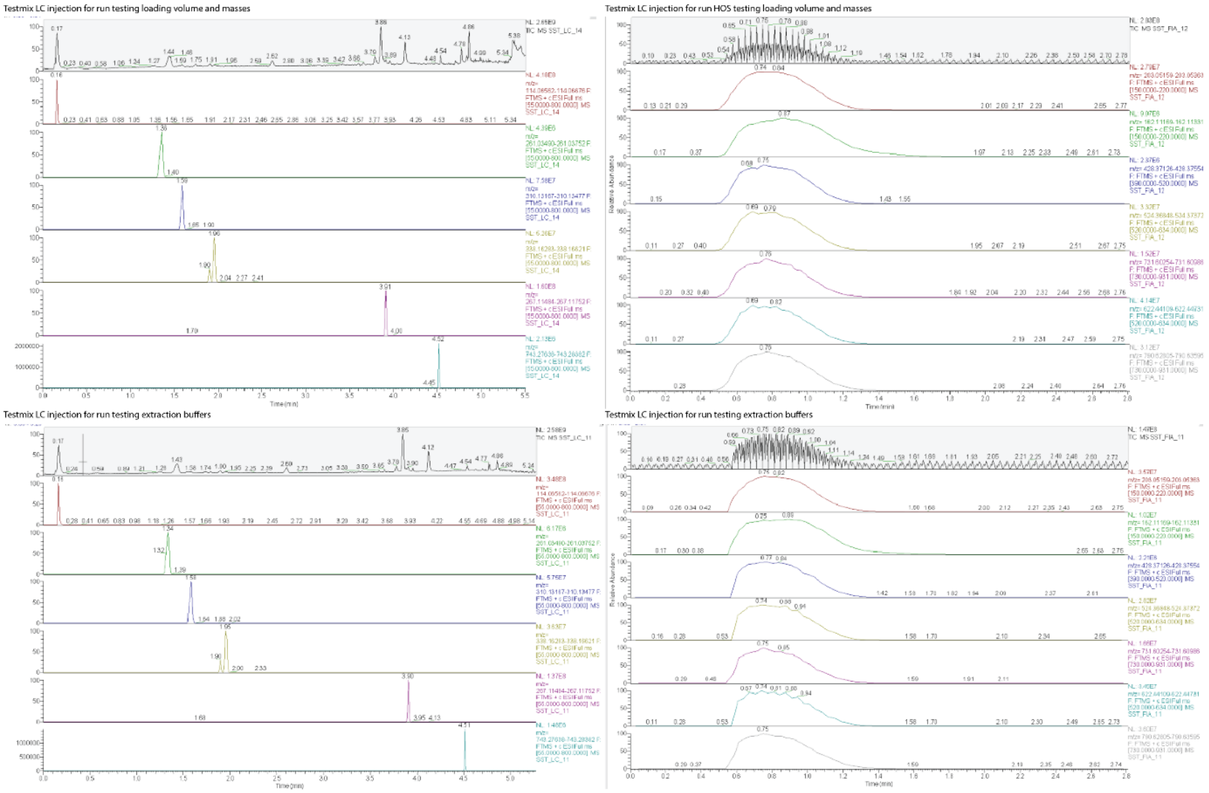

Supplementary Figure 3. System suitability test of test mix for liquid chromatography (LC, left) and flow injection analysis (FIA, right
